# PGPR-induced regulation of Zn and Fe transporters in wheat (*Triticum aestivum* L.) uncovered through integrated genome-wide analysis and functional validation

**DOI:** 10.1186/s12870-026-09415-8

**Published:** 2026-07-14

**Authors:** Faiza Maqbool, Rubab Zahra Naqvi, Raheela Rehman, Imran Amin, Ifrah Imran, Asma Imran

**Affiliations:** 1 Microbial Ecology Lab, Soil and Environmental Biotechnology Division, National Institute of Biotechnology and Genetic Engineering (NIBGE) a constituent college of PIEAS, P.O.Box 577, Faisalabad, Pakistan; 2 Molecular Virology and Gene Silencing Lab, Agricultural Biotechnology Division, NIBGE, National Institute of Biotechnology and Genetic Engineering (NIBGE) a constituent college of PIEAS, P.O.Box 577, Faisalabad, Pakistan; 3 Department of Plant Breeding and Genetics, University of Agriculture, Faisalabad, 38000 Pakistan

**Keywords:** Micronutrient, Biofortification, Genome-wide analysis, Wheat, Nutrient transporter, Zn and Fe homeostasis, Rhizobacteria nutrient mobilization, Plant growth promoting rhizobacteria

## Abstract

Zinc (Zn) and iron (Fe) deficiencies affect more than two billion people globally, particularly in cereal-dependent regions where wheat, despite its high consumption, provides inadequate micronutrient levels. Conventional interventions such as genetic modification and mineral supplementation remain costly, unevenly accessible, and insufficient for large-scale nutritional improvement. Agronomic biofortification using plant growth-promoting rhizobacteria (PGPR) offers a promising yet underexplored alternative, especially for regulating metal homeostasis genes in wheat. This research integrates multi-season field trials of Zn-biofortified Akbar-19 and the local cultivar Khaista-17, conducted under reduced fertilizer conditions with PGPR consortia. Afterwards, genome-wide analyses including phylogenetic relationships, promoter elements, gene interaction networks, expression profiles, and conserved domains/motifs of the *TaNAS* (19 genes), *TaNAAT* (6), *TaDMAS* (3), and *TaVIT* (31) gene families was performed. This was followed by transcriptional expression (qPCR) of six candidate genes in wheat grown under hydroponic Zn/Fe stress in the presence of PGPR. Field evaluation showed that PGPR inoculation boosted yield by 15–18% and increased grain Zn/Fe by 15–20% in Akbar-19 and 25–28% in Khaista-17, consistently outperforming fertilizer-only controls across both seasons. The genome-wide analyses exhibited the phylogenetic relationship of wheat *TaDMAS*, *TaNAAT*, and *TaNAS* genes with barley, while *TaVIT* and *TaVTL* genes with rice and maize. Promoter analyses of these genes showed an enrichment of stress-responsive cis-elements, such as IDE1/2, ZDRE1/2, IRO2-binding sites, and metal-responsive elements suggesting coordinated regulation of micronutrient chelation, uptake, and homeostasis. qPCR results confirmed PGPR-induced upregulation of NAS1, NAS6, NAS9, NAAT2, DMAS1, and VIT2 under Zn/Fe stress, with stronger induction in Khaista-17. Overall, the results show that PGPR modulate metal‑transporter gene networks and improve micronutrient biofortification in wheat, providing a genotype‑responsive and sustainable approach to address micronutrient deficiency.

**Supplementary Information:**

The online version contains supplementary material available at https://doi.org/10.1186/s12870-026-09415-8.

## Background

Zinc (Zn) and iron (Fe) deficiencies are persistent global health challenges, affecting over two billion individuals worldwide. According to the World Health Organization, approximately 30% of the global population is at risk of zinc deficiency [1]. Additionally, Fe deficiency remains the primary cause of anemia, impacting nearly 40% of pregnant women and 43% of children under the age of five [2]. These deficiencies are most widespread in Southeast Asia, Africa, and the Eastern Mediterranean, where limited dietary diversity and restricted access to supplementation make populations particularly vulnerable [3]. Despite the well‑established importance of these micronutrients for health, large segments of the global population especially in the developing countries still fail to meet recommended dietary allowances (RDA). Furthermore, dietary diversification and supplementation remain out of reach for many [4]. Hence, sustainable agricultural approaches are increasingly recognized as key to tackling micronutrient deficiencies at population scale [5, 6].

Wheat, the second staple, with a global output of 799 million tonnes in 2024-25 [7], plays a vital role in daily nutrition, by contributing ≈ 24% of total caloric intake, 33% of protein, and 3.5% of fat [8]. Globally, wheat contributes an estimated 6.49 mg of Fe and 4.69 mg of Zn per capita per day. However, intake levels are substantially lower in Asia. In South‑East Asia, wheat provides only 2.60 mg Fe and 1.88 mg Zn per capita, while South Asia shows slightly higher values at 6.72 mg Fe and 4.85 mg Zn [9]. The standard reference concentration in wheat grain declared by the US Department of Agriculture is 36 mg kg^− 1^ Fe and 26 mg kg^− 1^ Zn. To meet this nutritional needs, HarvestPlus has set the breeding target of 30 mg kg^− 1^ for Zn and 59 mg kg^− 1^ for Fe [10]. Despite its dietary significance, wheat does not sufficiently meet the RDAs for Fe and Zn [11]. This underscores the need for agronomic interventions emphasizing sustainable biofortification strategies in wheat [5]. A biologically driven approach based on soil microbiota, particularly rhizosphere and endophytic microbes with the ability to mobilize the soil metals, improve the plant nutrient uptake, and regulate the expression of plant transporters responsible for Zn and Fe distribution within grain has been reported recently [12, 13].

Under micronutrient deficiency, wheat acquire Fe and Zn through Strategy II which is a chelation-based mechanism involving phytosiderophores (PS), primarily mugineic acid (MA) and nicotianamine (NA). PS are secreted into the rhizosphere via the MA transporter TOM1, where they chelate Fe³⁺ and Zn²⁺ to form soluble complexes that are subsequently taken up by the roots. After Fe and Zn are released into the root cytosol, PS molecules are released into the rhizosphere for further chelation [14]. *Triticum aestivum* nicotianamine synthase (*TaNAS)*, nicotianamine aminotransferase (*TaNAAT)*, and deoxymugineic acid synthase ***(****TaDMAS)* are the core genes for PS biosynthesis, and their expression is modulated by the relative availability of Fe³⁺, Fe²⁺, and Zn²⁺ [15]. NA also functions as an intracellular chelator and transporter of Fe, Zn, Mn, and Cu, and serves as a precursor for deoxymugineic acid (DMA) synthesis in wheat and other graminaceous plants [16]. Although, the expression of PS-biosynthetic genes under micronutrient deficiency has been reported, their regulation in response to plant growth-promoting rhizobacteria (PGPR) remains poorly understood.

Genome-wide analyses revealed 21 *TaNAS*, 6 *TaNAAT*, and 3 *TaDMAS* genes in wheat exhibiting high sequence homology with their barley counterparts. Comparative expression analyses in wheat and barley demonstrate a conserved regulatory pattern, marked by strong induction of NAS, NAAT, and DMAS under Fe-deficient conditions and clear repression under Fe-sufficient conditions [15]. Similarly, under combined Zn–Fe deficiencies, these genes are upregulated to maintain the metabolite flux required for phytosiderophore biosynthesis in wheat. In bread wheat, 2′-DMA is synthesized via the methionine salvage pathway which is initiated with methionine being converted to S-adenosylmethionine (SAM). NAS then utilize three SAM molecules to generate one molecule of nicotianamine (NA) which is subsequently converted into a 3′′-ketointermediate by NAAT. DMAS catalyzes the final reduction step to produce DMA, completing the biosynthetic route essential for Fe and Zn acquisition [17].

Although, the response of transporter gene to micronutrient stress has been well characterized, genome-wide analyses that integrate stress-responsive expression with microbe-mediated nutrient mobilization remain limited. This knowledge gap constrains the development of sustainable strategies for crop micronutrient biofortification and soil health improvement. We hypothesized that PGPR inoculation will help in uptake of micronutrients and the expression of micronutrient transporters. The present study evaluated the PGPR-inoculation response of wheat genotypes under field conditions and a comprehensive assessment of the *TaNAS*, *TaNAAT*, *TaDMAS*, and *TaVIT* gene families, phylogeny, promoter features, gene structure, conserved domains, expression dynamics, and interaction networks. These analyses collectively advance our understanding of transporter functions in micronutrient acquisition and homeostasis. Furthermore, transcriptional profiling of *NAS*, *NAAT*, *DMAS*, and *VIT* genes in wheat shoots under controlled conditions demonstrates that PGPR inoculation modulates their expression in response to Fe and Zn deficiencies, likely by enhancing micronutrient availability in the rhizosphere.

## Materials and methods

### Bacterial strains

The bacteria used in this study were previously isolated from rhizosphere of wheat and rice from different regions in Pakistan. Isolation was performed using the standard serial dilution plating technique [18]. The glycerol stocks of bacteria (R14A, RZ-7, ER-29, 5D, K-1) were obtained from the laboratory collection and revived on LB plates. These strains were previously characterized for PGPR traits identified through 16 S rRNA gene sequence analysis (Table 1**)**.

**Table 1:** Representative bacterial isolates with identified species and diverse host, along with characterized plant growth-promoting traits and reported references. Solubilization index (SI), Data are mean± standard deviation (*n* = 4), +++, strong activity; ++, moderated activity; -, no activity

Isolates	Identified species	Source & Host	Zn-solubilization		*P*-solubilization		Growth on NFM	Sidero-phore production	IAA produc-tion	Reference
			Halo zone (mm)	SI	Halo zone (mm)	SI				
R14-A	*Stenotrophomonas sp.*	Indigofera roots	21.05 ± 1.08	7.24 ± 0.62	11.82 ± 0.34	4.24 ± 0.46	+++	+++	+++	[63]
Rz-7	*Cronobacter sp.*	Cholistan desert, weed	18.26 ± 0.66	6.52 ± 0.37	11.26 ± 0.61	3.74 ± 0.51	+++	+++	+++	[63]
K-1	*Pseudomonas sp.*	Kallar grass	19.67 ± 0.56	6.84 ± 0.28	9.82 ± 0.52	3.12 ± 0.74	+++	++	+++	[64]
ER-20	*Azospirillum lipoferum*	Rice roots	13.45 ± 0.37	5.43 ± 0.56	-	-	++	+++	++	[63]
5D	*Serratia marcescens*	Root nodules of chickpea	23.33 ± 0.48	7.48 ± 0.33	11.63 ± 0.47	4.06 ± 0.27	+++	+++	++	[65]

#### Qualitative assay for plant growth-promoting traits

The bacteria were tested for various plant growth-promoting traits, including zinc and phosphate solubilization, nitrogenase activity, siderophore production, and indole-3-acetic acid (IAA) production. The zinc solubilization was tested on tris-minimal agar supplemented with D-glucose and insoluble zinc oxide (ZnO; 1 g L^− 1^) equivalent to 0.1% Zn, as zinc source. Freshly grown bacterial cultures were spot-inoculated in triplicate with a sterile loop on the Zn-solubilization media plates. Plates were incubated at 30 ± 2 °C for 7 days, and the zinc solubilization was observed as a clear halo area around the colonies. The colony and halo zone diameters (mm) were recorded to determine the zinc solubilization index (ZSI) [19]. Phosphate solubilization was assessed by spot inoculation of pure culture on the petri plates containing Pikoviskaya medium with 0.5% tricalcium phosphate (TCP) as the insoluble phosphorus source. The plates were incubated at 30 ± 2 °C for 6–7 days and halo zones around the colonies indicated phosphate solubilization. The solubilization index (SI) was calculated using the formula by D Paul and SN Sinha [20]. The potential of bacteria to fix nitrogen was analyzed qualitatively by bacterial growth on a semi-solid nitrogen-free medium [21]. The chrome azurol S (CAS) agar assay, as described by B Schwyn and J Neilands [22], was used to test siderophore production. CAS agar plates spot-inoculated with freshly grown bacterial isolates were incubated at 30 ± 2 °C for 5 days. The presence of yellow-orange halo zones around colonies indicated siderophore production. The colorimetric assay was used to quantify IAA production [23]. The isolates were inoculated into Luria Bertani (LB) broth supplemented with 0.1% tryptophan as an IAA precursor and incubated at 28 °C for 7 days at 140 rpm. After incubation, the cultures were centrifuged at 10,000 rpm for 10 min. Subsequently, 100 µL supernatant was added to 100 µL of Salkowski’s reagent and incubated in the dark at room temperature for 20 min. The appearance of pink-to-purple color exhibited the presence of IAA or its metabolites. The bacterial strains were maintained on nutrient agar slants and stored at 4 °C until further use.

#### Formulation of bacterial consortium

To formulate the bacterial consortium, bacterial strains were subjected to in vitro compatibility assay as described [24]. Each bacterial strain was spot-inoculated onto LB agar plate, with the other strains spread across the plates, followed by incubation at 28 ± 2 °C for 72 h. The absence of an inhibition zone between colonies indicated compatibility. The individual strains were then cultured in LB broth at 28 ± 2 °C for 24 h. The strain were mixed in 1:1 ratio to form the consortium (10^9^ CFU mL^− 1^). The bacterial consortium (30%) was inoculated into sterilized carrier material (sugarcane waste/ filter mud) to formulate a biofertilizer. This biofertilizer was used to coat the wheat seeds before sowing by simply wetting the seeds with water sprinkler and dusting the biofertilizer with constant shaking till a thin and uniform layer of biofertilizer is coated on to the seed.

### Field-based assessment of wheat in response to nutrient stress and PGPR application

#### Field location and soil physicochemical properties

A field experiment was conducted to assess the crop over two consecutive wheat seasons (2022–2023, 2023–2024). The experimental field is located in Faisalabad, Punjab, Pakistan (31º 25’ N latitude and 73º 05’ E longitude) at an altitude of 184 m. The climate of the region is semi-arid (steppe), with cold, dry winters and average low temperatures around 4 °C, and hot summers with high temperatures usually above 40 °C especially during May and June. The baseline features of the soil at experimental site were EC = 0.84 dS m^− 1^, pH = 7.8, loamy in texture, organic matter = 1.08%, and low DTPA- extractable Zn = 0.42 mg kg^− 1^. The fertilizer doses applied (per hectare) was 130 kg N, 83.8 kg P, and 67.5 kg K. The crop was sown during November 25–28 and harvested during May 17–20 in both years.

#### Experimental design and treatments

The following five treatments were evaluated for the experiments:

I. F100: 100% fertilizer, i.e., recommended doses of N, P, and K.
II. F70: 70% fertilizer of the recommended doses of N, P, and K.
III. F70 + Zn/Fe: 70% NPK fertilizer + Zn (22 kg ZnSO_4_.7H_2_O) + Fe (8 kg FeSO_4_.7H_2_O) ha^-1^.
IV. F70 + B: 70% NPK fertilizer + Bacterial consortia.
V. F70 + B + Zn/Fe : 70% NPK fertilizer + Bacterial consortia + Zn (22 kg ZnSO_4_.7H_2_O) + Fe (8 kg FeSO_4_.7H_2_O) ha^-1^.

Two wheat genotypes, Akbar-19 and Khaista-17, were selected for these experiments. Akbar-19 is a high-yielding, rust-resistant wheat variety, known for improved tillering and medium height. It is a biofortified, zinc-enriched wheat variety. Khaista-17 is a tall, high-tillering, heat-stress-tolerant, low-Zn wheat variety. The seeds were obtained from Ayub Agriculture Research Institute (AARI) and the Department of Plant Breeding and Genetics at the University of Agriculture, Faisalabad. The experiment was arranged in a two-factorial randomized complete block design with 2 wheat genotypes and five treatments as factors, following three replicates per treatment. The plot size per replicate was 2m^2^ and wheat was sown using the dibbler method. The basal dose was applied before sowing as: full Zn, P, and K, 1/3 N were applied to the soil as per wheat crop recommendations. The remaining 2 doses of N were applied during the first and second irrigations. Bacterial-coated seeds were prepared one hour before sowing.

#### Morphological and agronomic attribute evaluation

During the vegetative stage, the plants were uprooted to evaluate morphological traits, including shoot fresh weight, root fresh weight, number of tillers, shoot length, and root length. At physiological maturity, plant height was recorded manually from the ground level to the apex of the spike using a meter rod. After harvesting, the number of fertile tillers, spike length, and spike weight alongwith the total biomass, grain weight, and 1000-grain weight were measured to assess crop performance and yield potential. Data was collected from three plants per replicate, with three replicates conducted per treatment.

#### Grain nutrient analysis

Grains at physiological maturity were collected and packed in labelled polythene zipper bags. The grains were then dried in the oven at 60± 2 °C in airtight conditions for 72 h. A stainless-steel grinder was used to finely grind the wheat grains. To analyze the nutrients, 1 g of each sample was weighed and digested with 10 mL of a di-acid mixture (HNO_3_:HClO_4_) in a 9:4 ratio at 300 °C using a digestor until the solution became colorless. The digested samples were transferred to a 100mL volumetric flask, and the volume was adjusted to 100mL with distilled water. The diluted samples were subjected to phosphorus (P) quantification using a spectrophotometer (Camspec M-350 double-beam UV visible spectrophotometer, United Kingdom) and to Zn and Fe analysis using atomic absorption spectrophotometry (Agilent AA240FS, Fast sequential atomic absorption spectrophotometer), along with standards [25].

#### Estimation of Zn and Fe use efficiency

The Zn and Fe use efficiencies was determined using the formulas given by [26] to calculate estimated values.

$$\:\mathrm{Z}\mathrm{n}\:\mathrm{u}\mathrm{s}\mathrm{e}\:\mathrm{e}\mathrm{f}\mathrm{f}\mathrm{i}\mathrm{c}\mathrm{i}\mathrm{e}\mathrm{n}\mathrm{c}\mathrm{y}\:=\left({\mathrm{G}\mathrm{Y}}_{zn}\:\mathrm{―}\:{\mathrm{G}\mathrm{Y}}_{c}\right)/{\mathrm{Z}\mathrm{n}}_{a}\:$$

$$\:\mathrm{F}\mathrm{e}\:\mathrm{u}\mathrm{s}\mathrm{e}\:\mathrm{e}\mathrm{f}\mathrm{f}\mathrm{i}\mathrm{c}\mathrm{i}\mathrm{e}\mathrm{n}\mathrm{c}\mathrm{y}\:=({\mathrm{G}\mathrm{Y}}_{Fe}\:\mathrm{―}{\mathrm{G}\mathrm{Y}}_{c})/{\mathrm{F}\mathrm{e}}_{a}\:$$

Where GY_Zn_ is the grain yield of Zn fertilized plots (kg ha^− 1^), GY_Fe_ is the grain yield of Fe fertilized plots (kg ha^− 1^), GY_c_ is the grain yield of unfertilized plots (kg ha^− 1^), Zn_a_ is the amount of Zn fertilizer (kg ha^− 1^) and Fe_a_ is the amount of Fe fertilizer (kg ha^− 1^) applied.

#### Statistical analysis

Two-way ANOVA was used to analyze the data, with two factors (wheat genotype and treatment). The pairwise comparisons were used to compare the means of the main effects (genotype and treatment) and the interaction effects (genotype x treatment) and to test them using the least significant difference (LSD) test a significance level of 5%. The Pearson correlation analysis (was performed utilizing IBM SPSS Statistics 20 software to evaluate the relationships among agronomic traits. The resulting correlation matrix was manually visualized as a heatmap using Microsoft powerpoint, employing conditional formatting with a three-color scale to delineate the strength and direction of associations. The asterisk included to the heatmap indicates the level of significance (*p *≤ 0.05).

### Genome-wide analysis

The protein sequences of the *TaDMAS*, *TaNAAT*, *TaNAS*, and *TaVIT* gene families in wheat, rice, barley, maize, soybean, and Arabidopsis were obtained from the NCBI, Phytozome, and Ensembl Plants databases.

#### Phylogenetic analysis

To infer the phylogenetic tree, MEGA 11 software was used, and further annotation and visualization was performed using iTol software to understand the evolutionary relationships among the *TaDMAS*, *TaNAAT*, *TaNAS*, and *TaVIT* gene families in wheat, rice, barley, maize, soybean, and *Arabidopsis*. Phylogenetic relationships were inferred from a bootstrap consensus tree, which was considered accurate and credible.

#### Promoter analysis

The promoter analysis was performed to annotate and visualize the cis-regulatory sequences within 1.5 kb promoters of *TaNAS*, *TaNAAT*, *TaDMAS*, and *TaVIT* genes. All the genes were annotated using Geneious Prime. The cis-acting elements included the *TATA-Box* (TATA), iron deficiency-responsive elements (IDE1 and IDE2), zinc deficiency responsive element (*ZDRE-1* and *ZDRE-2*), Iron-related transcription factor -binding sequence (*IRO2-BS*), and metal-responsive elements (*MRE*). These elements are involved in the regulation of genes related to iron and zinc metabolism.

The annotation of cis-acting elements within the 1.5 kb promoter regions of the *TaNAS*, *TaNAAT*, *TaDMAS1* and *TaVIT* genes included six key promoters: *TATA-Box* (TATA), *IDE1* (5′-ATCAAGCATGCTTCTTGC − 3′), *IDE2* (5′-TTGAACGGCAAGTTTCACGCTGTCACT-3′), *ZDRE-1* (5′- ATGTCGACAT-3′), *ZDRE-2* (5′- ATGTCGACAC-3′), *IRO2-* binding sequence (5′-CACGTGG-3′) and *MRE*-like element (5′-TGCAGGC-3′). Furthermore, the *IDE1* and *IDE2* elements, which are responsive to iron-deficiency, were annotated within the 1.5 kb promoter regions of genes exhibiting 60% sequence similarity. *ZDRE 1* and *ZDRE 2* elements were identified with no more than one mismatch to the consensus sequence (RTGTCGACAY) [27]. Additionally, analyses revealed the presence of core iron-related transcription factor-binding sites (*IRO2-BS*) and metal-responsive elements (*MRE*-like element), within the 1.5 kb promoter regions, exhibiting 100% similarity identity.

#### Gene interaction and pathways analysis

The protein sequences of *TaDMAS*, *TaNAAT*, *TaNAS*, and *TaVIT*, retrieved from Ensemble Plants, NCBI, and Phytozome, were subsequently fed into STRING 11.0 to construct the interaction network of the selected genes. To gain a more comprehensive understanding of the pathway interactions among the studied gene families, an online tool, Shiny GO vo.8, was used. The retrieved gene IDs of the selected genes were uploaded to this web server to generate a pathway interaction dot plot.

#### Gene structure and domain analyses

For gene structure analysis, the genomic and CDS sequences were retrieved from databases and analyzed using the online Gene Structure Display Server (GSDS). For domain analysis, the retrieved protein sequences of selected genes were uploaded to the Conserved Domain Database (CDD-NCBI), and the resulting data were further annotated using TB Tools-II software.

#### Conserved motif analysis

The conserved motifs of protein sequences was predicted using MEME (Multiple Expectation Maximization for Motif Elicitation) suite (version 5.5.5) web server. The motifs width was set to range from 5 to 200 amino acids, with the number of motifs specified as 10 for *TaDMAS*, *TaNAAT* and *TaNAS*, and 12 motifs for *TaVIT*.

#### *In*-*silico* expression analysis

For in-silico expression analyses, a total of 31 *TaVIT*, 19 *TaNAS*, 6 *TaNAAT*, and 3 *TaDMAS* genes were selected. The wheat expression browser, exp Vip was used to retrieve the expression values in the form of TPMs. The extracted data were used to generate an expression heatmap using TBtools-II software. The absolute values were used to illustrate the developmental and tissue-specific expression patterns.

### PGPR-mediated expression analysis of Zn and Fe transporters in wheat

#### Set up of hydroponic experiment

A hydroponic experiment was conducted to evaluate the effects of inoculation on Zn and Fe transporters in wheat plants under Zn and Fe-deficient conditions in a greenhouse environment at NIBGE, Faisalabad. Seeds were surface sterilized using 2% sodium hypochlorite for 10 min, followed by successive rinses (5–6 times) with sterile distilled water. The broth cultures of five PGPR strains were grown separately to reach 10^9^ CFU mL^− 1^. The sterilized seeds were primed with PGPR by soaking the seeds in a PGPR consortium for 30 min. Control seeds were treated with sterile nutrient broth. Both inoculated and uninoculated seeds were germinated on moist, sterile filter paper, under a relative humidity of 70–80% and day/night temperature of 25 °C /20°C in a greenhouse setting. After four days, the wheat seedlings were shifted to a Phytabox with a volume of 350 mL, filled with half-strength Hoagland solution.

The experiment was arranged in a two-factorial completely randomized design (CRD). A total of three replicates were maintained for seven treatments. Twenty seedlings per Phytabox were grown under a daily temperature range of 25 ± 2 °C during the day and 20 ± 2 °C at night, with a photoperiod of 14-h light and 10-h darkness. The detailed treatments for the hydroponic experiment are listed in Table 2. The pH of the Hoagland solution was maintained at 6.5 ± 0.5 before its inoculation into the Phytabox and aeration was maintained to ensure supply of dissolved oxygen. The Hoagland solution for each treatment was changed twice weekly. The plants were harvested four weeks after cultivation in a hydroponic system, and the three replicates from each treatment were used for expression studies of Zn and Fe transporter genes.

**Table 2:** Treatment outline for the hydroponic experiment

Treatment		Hoagland solution			Bacterial treatment
		Fe (gL^− 1^)	Zn (mgL^− 1^)	Details	
C	Positive control	0.74	0.59	Half strength with ZnCl_2_ and FeEDTA (Fe and Zn-supplemented)	Un-inoculated
T1	Negative control-1	0.16	**-**	Half strength with insoluble Fe_2_O_3_ (Fe-deficient)	Un-inoculated
T2	Consortia-inoculated	0.16	**-**	Half strength with insoluble Fe_2_O_3_ (Fe-deficient)	Inoculated
T3	Negative control-2	**-**	0.13	Half strength with insoluble ZnO (Zn-deficient)	Un-inoculated
T4	Consortia-inoculated	**-**	0.13	Half strength with insoluble ZnO (Zn-deficient)	Inoculated
T5	Negative control-3	0.16	0.13	Half strength with insoluble Fe_2_O_3_ and ZnO (Fe and Zn-deficient)	Un-inoculated
T6	Consortia-inoculated	0.16	0.13	Half strength with insoluble Fe_2_O_3_ and ZnO (Fe and Zn-deficient)	Inoculated

#### Validation of qPCR for expression of Zn and Fe transporter genes in the shoot of contrasting wheat genotypes

The expression of genes (*NAS1*, *NAS6*, *NAS9*, *NAAT2*, and *DMAS1*) and vacuolar iron transporter genes (*VIT2*) involved in phytosiderophore production pathway, which potentially play roles in Zn and Fe chelation and transport, were selected for validation via RT-PCR under various treatments of Fe and Zn-deficiency conditions. Total RNA was extracted from shoot samples, followed by DNase I treatment (using Thermo Scientific) to remove genomic DNA contamination. The complementary DNA (cDNA) was synthesized from 1 µg (1 µg) of total RNA using the high-capacity cDNA reverse transcription kit (Applied Biosystems™) and an oligo (dT) primer. PCR was performed in 96-well plates using the PowerUp™ SYBR™ Green Master Mix (Applied Biosystems™). The gene-specific primers for Zn and Fe transporter genes (Table 3**)** facilitated the assessment of differential gene expression in selected wheat varieties. The Actin gene was used as an endogenous reference for normalizing the results. Three independent biological replicates, each with three technical replicates, were maintained for each cDNA sample. Relative gene expression levels were calculated using the 2-^ΔΔCt^ method and the statistical differences of the mean differences were evaluated using Tukey’s test (*P* < 0.05 & *P* < 0.01).

**Table 3:** Studied *TaNAS*, *TaNAAT*, *TaDMAS*, and *TaVIT* gene and their primer sequences used for qPCR analysis

Gene name	Identifiers/ IWGSC Gene Id	Chromosome Location	Forward Primer	Reverse Primer	References
TaNAS1-A	Traes_2AS_1FB8F6760.1/ KU529948.1	2AS	AAGTCGACGCGCTCTTCACC	GCTCAGGTTGATGTAGTTGTCA	[49]
TaNAS6-D	Traes_4DL_AF0869DDB.1/ KU529961.1	4DL	GACGACGACGTGGTGAACT	GATCAGAAGGCCACTTCA	
TaNAS9-D	Traes_2DS_FE40FC64C.1/ KU529953.1	2DS	GCCTCCTTCGACAACTACGA	ACCACCGGGTACAGGAAG	
TaNAAT2-B*	Traes_1BL_D8276D3DB/ TraesCS1B02G300600.1	1BL	AATTGGAGGTTCGACATTTTG	CTTTCTTGCCACCTCTGCAA	[57]
TaDMAS1-A	Traes_4AS_887399584/KX348551.1	4AS	CGTCCAGGGGCAAGAGTG	GAGCTCCTCGAGGGACTTGT	
TaVIT2-5 A	TRIAE_CS42_5AL_TGACv1_374137_AA1191460/ TraesCS5A02G203400.1	5BL	CTCCCCCTACATGTTCGT	CCCTTGACGTAGCCGAA	[53]

## Results

### Bioassay for PGP activity profiling of rhizobacterial isolates

**Fig. 1:**
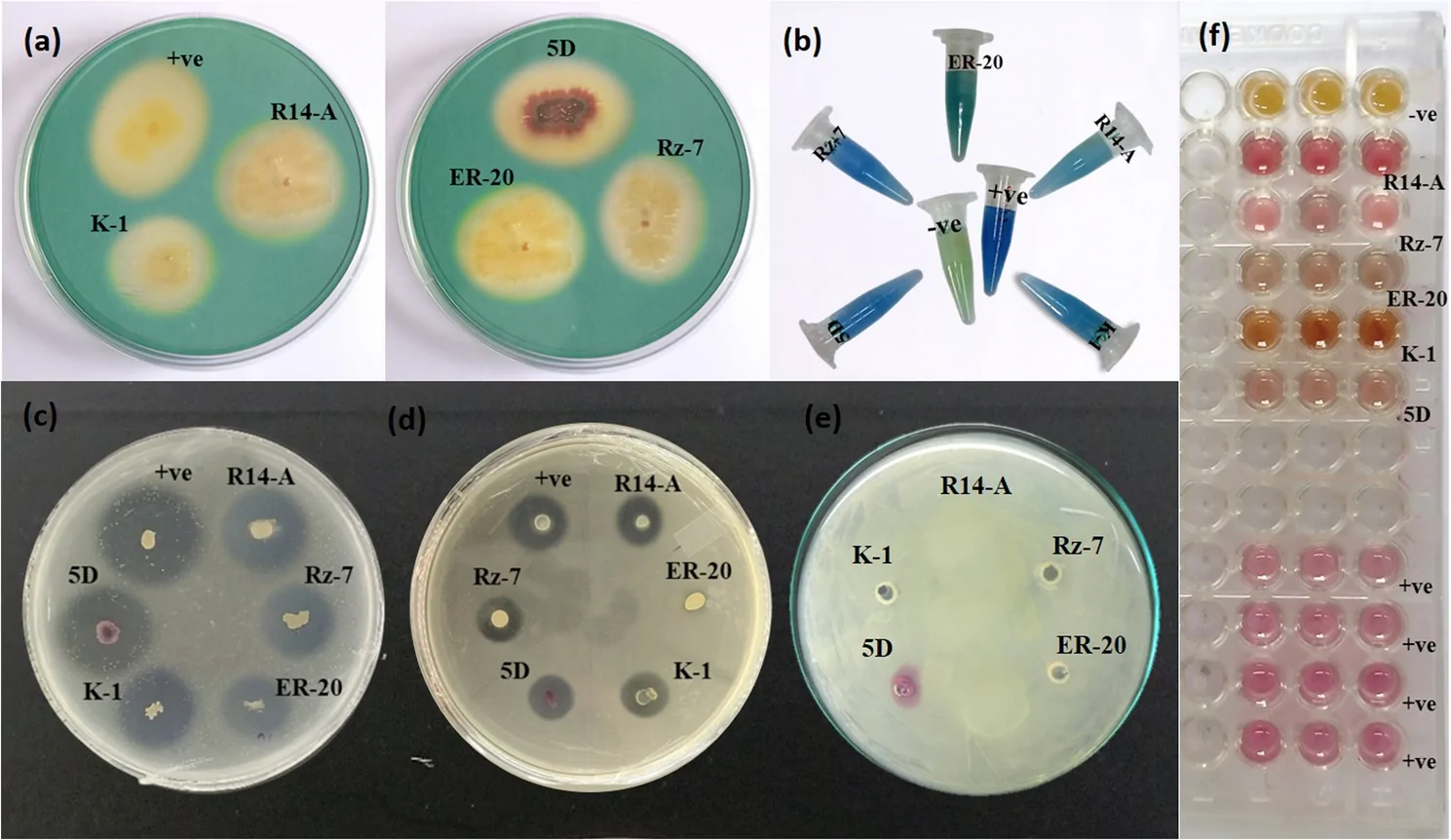
(**a**) Siderophore production was detected by formation of light orange halo zones on CAS agar plates, (**b**) Nitrogen fixation was confirmed by blue color development of isolates in NFM after 5 days incubation, (**c**) Zinc and (**d**) phosphorus solublization was tested by formation of halo zone on an agar plate supplemented with Zinc oxide (ZnO) and tri-calcium phosphate (TCP), (**e**) In-vitro compatibility was tested with no zone of inhibition after 5 days incubation, (**f**) IAA production was detected by pink coloration upon reaction with Salkowski’s reagent

The data for the PGP traits (Fig. 1; Table 1) show that all bacteria solubilized ZnO, with 5D and R14-A showing the maximum solubilization, followed by K-1, Rz-7, and ER-20 (7.48, 7.24, 6.84, 6.52, 5.42, respectively) Fig. 1**-(c**,** d)**. Of the five rhizobacteria, four showed the potential for phosphorus solubilization with phosphorus solubilization index (PSI) values of R14-A (4.28), 5D (4.06), Rz-7 (3.74), and K-1 (3.12) (Table 1**)**. The nitrogenase activity assay on (NFM) showed that R14-A, Rz-7, ER-20 and 5D exhibited the developed a blue color, indicating their potential for nitrogen fixation **(**Fig. 1-b**)**. The siderophore assay (Fig. 1-a**)**, showed siderophore production, with R14-A, Rz-7, ER-20, and 5D showing the maximum chelation. All 5 isolates produced IAA, as indicated by the appearance of pink color (Fig. 1-f**)**. IAA production was estimated as dark pink coloration by R14-A, Rz-7, and K-1, and as light pink coloration by ER-20 and 5D Table 1.

### Field assessment of wheat genotypes

#### Comparative analysis of treatment and genotype effect on morphological and vegetative traits

A noticeable genotypic and treatment-dependent response was observed across all studied traits, indicating a differential response of genotypes to fertilizer treatments and bacterial inoculation (Table 4). During the vegetative stage, both Akbar-19 and Khaista-17 genotypes were notably affected by reduced NPK fertilization, with or without Zn supplementation. In the 2022-23 season, Akbar-19 exhibited significant improvement in SFW (30.6 g), RFW (3.3 g), NT (14 tillers), SL (83.3 cm), and RL (19 cm) under F70 + B + Zn/Fe, closely followed by F70 + B treatment, suggesting a synergistic effect of bacterial inoculation on plant biomass and shoot development. Notably, both treatments outperformed the control F100 in terms of effectiveness. Similar responses were observed in Khaista-17, where F70 combined with B and Zn/Fe resulted in higher tillers (12 tillers), SFW (30.2 g), RFW (3.0 g), SL (77.3 cm), and RL (17.8 cm). In (2023–24), Akbar-19 consistently reproduced the genotype-treatment response, achieving the highest SFW (30.5 g), RFW (3.3 g), NT (13 tillers), SL (80.3 cm), and RL (17 cm) under F70 + B + Zn/Fe, thereby confirming the seasonal reliability of this treatment. Likewise, Khaista-17 produced the highest NT (12 tillers), SFW (29.4 g), RFW (3.2 g), SL (75.7 cm), and RL (16 cm) under the same treatment. Over two consecutive years, F70 + B + Zn/Fe and F70 + B demonstrated superior efficacy compared to other treatments, resulting in significant enhancements in early vegetative growth as well as yield traits. Conversely, F70 alone resulted in reduced trait expression, highlighting the importance of bacterial and micronutrient synergy for robust morphological growth under reduced fertilizer application.

**Table 4:** Mean of morphological traits at vegetative and yield stage affected by F100 (100% fertilizer, i.e., recommended dose of N, P, and K), F70 (70% fertilizer of the recommended dose of N, P, and K), Zn/Fe, and bacterial consortia (B). The table shows mean values (of 3 replicates), ± Standard deviation. Different letters signify statistically significant differences, and identical letters signify statistical similarity determined by the least significant differences (LSD) test at a 0.05 probability level. **SFW**, Shoot fresh weight (g); **RFW**, Root fresh weight (g); **NT**, No. of Tillers; **SL**, Shoot length (cm); **RT**, Root length (cm); **NFT**, No. of Fertile Tillers; **SL**, Shoot length (cm); **SpL**, Spike Length (cm); **SpW**, Spike weight (g)

Year 2022-23										
Variety	Treatment	Vegetative stage					Yield stage			
		SFW	RFW	NT	SL	RT	NFT	SL	SpL	SpW
Akbar-19	F100	26.3 ± 1.01 ^bc^	2.9 ± 0.14 ^abc^	12.0 ± 0.84 ^abcd^	77.0 ± 1.23 ^bc^	16.3 ± 0.78 ^abc^	9.0 ± 0.84 ^abc^	93.7 ± 0.97 ^bc^	11.4 ± 0.30 ^ab^	18.7 ± 1.08 ^ab^
	F70	21.6 ± 0.61 ^e^	2.4 ± 0.09 ^d^	8.8 ± 0.58 ^ef^	72.7 ± 1.42 ^cd^	14.3 ± 0.63 ^cd^	6.2 ± 0.35 ^de^	88.7 ± 1.42 ^cde^	9.2 ± 0.30 ^cd^	15.0 ± 0.78 ^cde^
	F70 + Zn/Fe	24.5 ± 0.62 ^cd^	2.6 ± 0.11 ^bcd^	10.7 ± 0.71 ^cde^	74.7 ± 1.63 ^cd^	15.7 ± 0.71 ^abcd^	7.7 ± 0.71 ^bcde^	90.7 ± 1.63 ^cde^	10.5 ± 0.56 ^bc^	16.6 ± 0.89 ^bcde^
	F70 + B	27.9 ± 0.98 ^ab^	3.1 ± 0.05 ^ab^	13.5 ± 0.40 ^ab^	81.0 ± 1.23 ^ab^	17.7 ± 0.71 ^abc^	8.8 ± 0.35 ^abc^	97.7 ± 1.63 ^ab^	12.1 ± 0.37 ^a^	18.5 ± 0.35 ^ab^
	F70 + B + Zn/Fe	30.6 ± 0.45 ^a^	3.3 ± 0.17 ^a^	14.2 ± 0.58 ^a^	83.0 ± 0.93 ^a^	19.0 ± 1.23 ^a^	10.2 ± 1.10 ^a^	101.3 ± 1.87^a^	12.6 ± 0.37^a^	20.8 ± 0.91 ^a^
Khaista-17	F100	26.3 ± 0.74 ^bc^	2.7 ± 0.12 ^bcd^	10.2 ± 0.81 ^de^	73.3 ± 0.97 ^cd^	15.8 ± 0.71 ^abcd^	7.8 ± 0.48 ^bcde^	87.7 ± 0.97 ^de^	11.5 ± 0.33^ab^	17.0 ± 1.17 ^bcd^
	F70	23.4 ± 0.62 ^de^	2.5 ± 0.06 ^cd^	7.7 ± 0.81 ^f^	67.0 ± 1.39 ^e^	12.5 ± 0.93 ^d^	5.7 ± 0.48 ^e^	81.7 ± 1.76 ^f^	8.8 ± 0.42 ^d^	13.8 ± 0.47 ^e^
	F70 + Zn/Fe	26.1 ± 0.72 ^bc^	2.9 ± 0.14 ^abc^	9.2 ± 0.35 ^ef^	70.3 ± 1.42 ^de^	14.8 ± 1.17 ^bcd^	6.8 ± 0.35 ^cde^	86.3 ± 1.42 ^ef^	10.3 ± 0.34 ^bc^	14.2 ± 0.60 ^de^
	F70 + B	28.6 ± 0.67 ^ab^	2.9 ± 0.12 ^abc^	11.2 ± 0.58 ^bcde^	75.3 ± 0.97 ^cd^	17.2 ± 0.97 ^abc^	8.2 ± 0.58 ^abcd^	91.3 ± 0.97 ^cde^	11.5 ± 0.19 ^ab^	17.5 ± 0.95 ^bc^
	F70 + B + Zn/Fe	30.2 ± 0.22 ^a^	3.0 ± 0.17 ^ab^	12.8 ± 0.58 ^abc^	77.3 ± 1.87 ^bc^	17.8 ± 0.71 ^ab^	9.7 ± 0.48 ^ab^	92.7 ± 1.49 ^bcd^	11.9 ± 0.35 ^a^	19.0 ± 0.58 ^ab^
Year 2023-24										
Akbar-19	F100	27.7 ± 0.52 ^cd^	2.9 ± 0.14 ^abcd^	10.8 ± 0.71 ^abcd^	74.3 ± 1.76 ^c^	14.7 ± 0.71 ^abc^	8.4 ± 0.71 ^abc^	91.3 ± 1.76 ^c^	10.9 ± 0.30 ^ab^	18.0 ± 0.32 ^ab^
	F70	24.5 ± 0.42 ^fg^	2.5 ± 0.15 ^de^	7.8 ± 0.35 ^e^	68.7 ± 0.97 ^de^	13.7 ± 0.71 ^bcd^	6.0 ± 0.48 ^d^	85.7 ± 0.97 ^de^	8.7 ± 0.30 ^cd^	15.7 ± 0.36 ^e^
	F70 + Zn/Fe	25.7 ± 0.42 ^ef^	2.8 ± 0.15 ^bcde^	8.7 ± 0.35 ^de^	71.7 ± 1.17 ^cd^	15.0 ± 0.46 ^ab^	6.9 ± 0.35 ^cd^	88.7 ± 1.17 ^cd^	10.0 ± 0.56 ^bc^	16.4 ± 0.41 ^de^
	F70 + B	29.2 ± 0.50 ^abc^	3.1 ± 0.11 ^ab^	11.7 ± 0.81 ^abc^	78.7 ± 0.97 ^ab^	16.0 ± 0.46 ^ab^	8.9 ± 0.81 ^abc^	95.7 ± 0.97 ^ab^	11.6 ± 0.37 ^a^	17.8 ± 0.46 ^bc^
	F70 + B + Zn/Fe	30.5 ± 0.61 ^a^	3.3 ± 0.21 ^a^	13.0 ± 0.61 ^a^	80.3 ± 0.97 ^a^	17.0 ± 0.46 ^a^	10.2 ± 0.61 ^a^	97.3 ± 0.97 ^a^	12.2 ± 0.37 ^a^	19.1 ± 0.31 ^a^
Khaista-17	F100	26.4 ± 0.54 ^de^	3.0 ± 0.06 ^abc^	10.2 ± 0.94 ^bcde^	69.0 ± 0.93 ^de^	13.7 ± 0.97 ^bcd^	7.9 ± 0.35 ^bcd^	86.0 ± 0.93 ^de^	11.1 ± 0.33 ^ab^	15.9 ± 0.24 ^e^
	F70	23.6 ± 0.39 ^g^	2.4 ± 0.10 ^e^	7.7 ± 0.48 ^e^	65.3 ± 0.71 ^e^	11.3 ± 0.71 ^d^	6.1 ± 0.25 ^d^	82.3 ± 0.71 ^e^	8.4 ± 0.42 ^d^	13.7 ± 0.24 ^f^
	F70 + Zn/Fe	24.9 ± 0.39 ^efg^	2.6 ± 0.03 ^cde^	9.3 ± 0.81 ^cde^	66.7 ± 0.97 ^e^	12.0 ± 0.93 ^cd^	6.9 ± 0.58 ^cd^	83.7 ± 0.97 ^e^	9.9 ± 0.34 ^bc^	14.1 ± 0.31 ^f^
	F70 + B	27.9 ± 0.32 ^bcd^	3.0 ± 0.12 ^abcd^	10.8 ± 0.71 ^abcd^	72.7 ± 1.17 ^cd^	15.3 ± 0.71 ^ab^	8.7 ± 0.46 ^abc^	89.7 ± 1.17 ^cd^	11.2 ± 0.19 ^ab^	16.6 ± 0.24 ^cde^
	F70 + B + Zn/Fe	29.4 ± 0.33 ^ab^	3.2 ± 0.10 ^ab^	12.2 ± 0.75 ^ab^	75.7 ± 1.17 ^bc^	16.0 ± 1.23 ^ab^	9.7 ± 0.70 ^ab^	92.7 ± 1.17 ^bc^	11.5 ± 0.35 ^a^	17.3 ± 0.54 ^bcd^

The yield-related morphological traits, mainly plant height (PH), number of fertile tillers (NFT), spike length (SpL), and spike weight (SpW) across both cropping seasons and genotypes, exhibited notable improvements under reduced fertilization regimes supplemented with bacterial consortia, regardless of Zn inclusion. In the 2022-23 season, Akbar-19 showed the highest NFT (10.2), PH (101.3 cm), SpL (12.6 cm), and SpW (20.8 g) under F70 + B + Zn/Fe, outperforming the F100 control. Khaista-17 responded similarly with NFT of 9.7, PH of 92.7 cm, SpL of 11.9 cm, and SpW of 19 g, signifying genotype-independent response to the treatment. Both genotypes maintained this trend in the successive years, confirming the reliability of the response. While Akbar-19 consistently outperformed Khaista-17 in PH and SpW, the genotypes showed statistically similar response concerning NFT and SpL.

#### Agronomic performance and grain nutrient accumulation in wheat genotypes

**Fig. 2:**
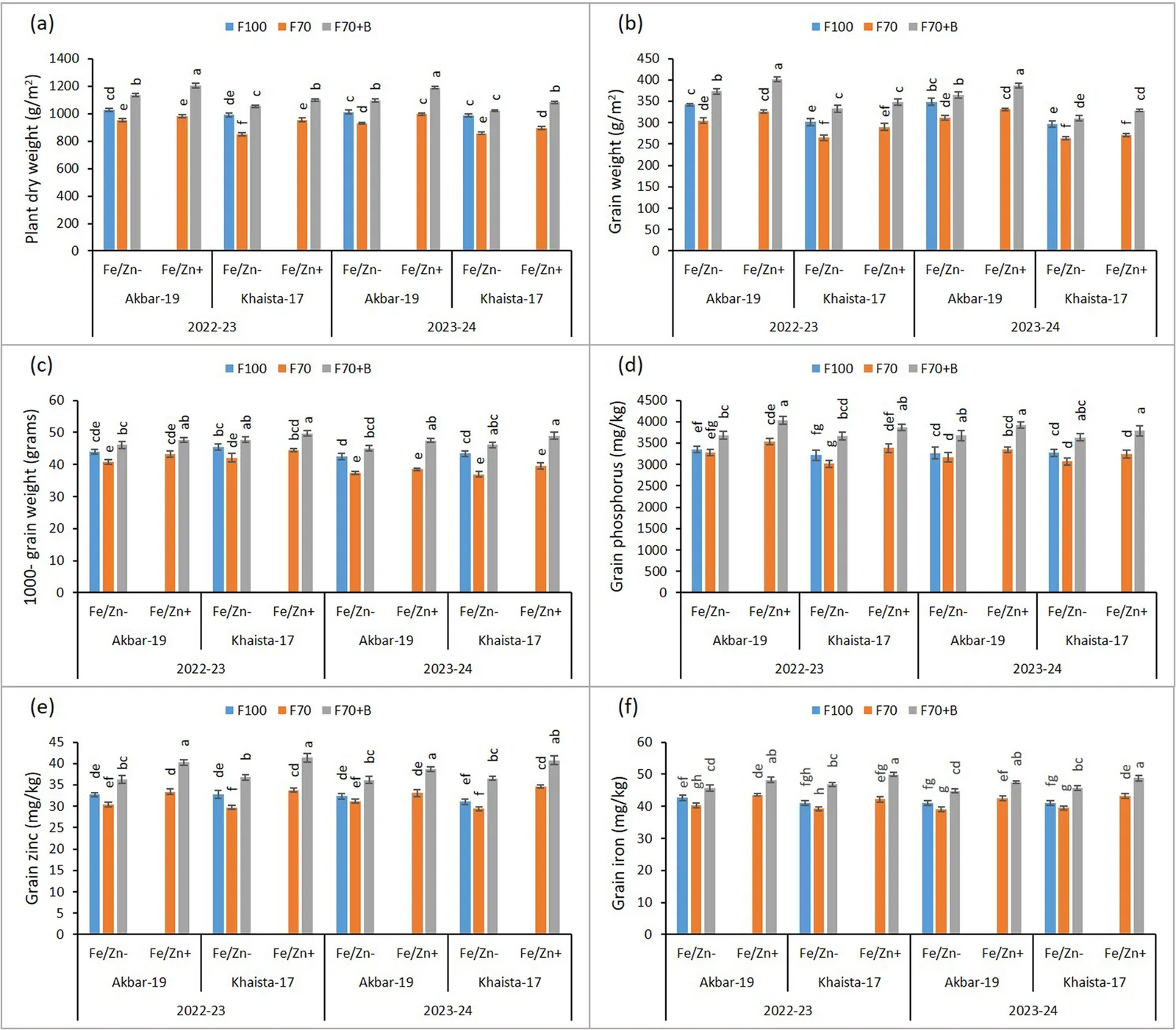
Effect of F100 (100% fertilizer i.e., recommended dose of N, P and K), F70 (70% fertilizer of recommended dose of N, P and K), Zn/Fe and bacterial consortia (B) on agronomic traits (**a**) Plant dry weight (g m^− 2^) (**b**) Grain weight (g m^− 2^) (**c**) 1000-grain weight (g) and nutrient content (**d**) Grain Phosphorus (mg kg^− 1^) (**e**) Grain Zinc (mg kg^− 1^); (**f**) Grain iron (mg kg^− 1^). Error bars represent standard error. Different letters signify statistically significant differences, and identical letters signify statistical similarity determined by the least significant differences (LSD) test at a 0.05 probability level

The key agronomic traits, primarily plant dry weight (PDW m^− 2^), grain weight (GW m^− 2^), and thousand grain weight (TGW) were assessed across the two genotypes and seasons as depicted in Fig. 2 (a), (b), and (c). Akbar-19 yielded the highest PDW (1203.6 g) and GW (401.2 g) under combined application of Zn/Fe and bacterial consortia at F70, thereby significantly surpassing the yield observed in F100 control. Furthermore, treatment F70 + B demonstrated a significant positive effect, with PDW of 1132.1 g, GW of 373 g, and TGW of 46.1 g, highlighting the efficacy of bacterial inoculation in enhancing the yield under nutrient stress conditions. Khaista-17 exhibited an improved TGW (49.7 g) and PDW (1097.6 g) under the treatment F70 + B + Zn/Fe, followed by F70 + B (TGW: 47.7 g, PDW: 1052.8 g). These performance patterns were consistently reproduced in the subsequent year (2023-24), confirming the reliability of the response. The F70 + B treatment yielded results as observed for other traits, whereas F70 with or without Zn underperformed, highlighting the significance of synergistic effect of microbial and nutrient inputs in maximizing crop productivity. Generally, Akbar-19 produced higher PDW and GW compared to Khaista-17, which exhibited higher TGW, thereby indicating a genotype-specific response.

The grain concentrations of phosphorus (P), zinc (Zn), and iron (Fe) were significantly influenced by bacterial inoculants and Zn/Fe fertilizer, exceeding the effects of synthetic, high-dose fertilizers. As shown in Fig. 2**(d)**,** (e)**,** and (f)**, under optimal treatment conditions, the two distinct genotypes showed consistent response across both seasons, exhibiting improved levels of grain P, Zn, and Fe, thereby facilitating efficient nutrient delivery. During the first year (2022-23), the most effective treatment combinations, F70 + B + Zn/Fe and F70 + B, yielded the highest grain Zn concentrations, 40.2 and 36 mg/kg in Akbar-19 and 41.4 and 36.8 mg/kg in Khaista-17, respectively, outperforming the control treatment F100. Figure 2(d) highlights the crucial role of bacterial inoculation for improved grain P content across two cropping seasons. The treatment F70 + B + Zn/Fe showed enhanced grain P reaching 4034 mg/kg in Akbar-19 and 3870 mg/kg in Khaista-17, followed by F70 + B. Furthermore, Khaista-17 exhibited the maximum grain Fe concentration (49.9 mg/kg), followed by Akbar-19 (48.2 mg/kg) during the 2022-23 season. Although slightly lower Fe content in Akbar-19, these finding are indicative of micronutrient biofortification facilitated by co-application of bacteria and Zn. Despite some variation, both genotypes consistently performed well across treatments, highlighting the strong stimulatory effect of the F70 + B + Zn/Fe regime.

#### Trait correlation analysis

**Fig. 3:**
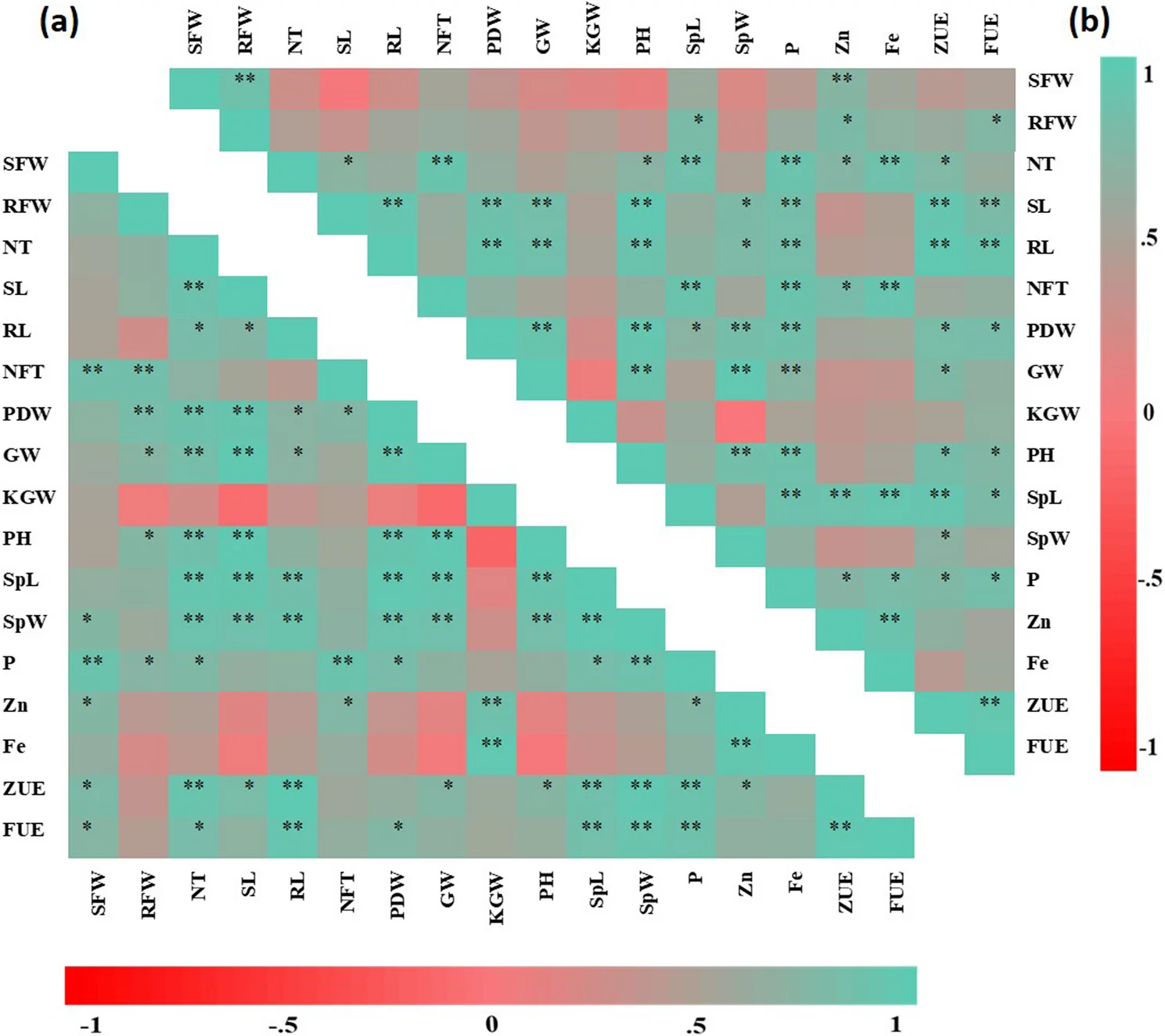
Heatmap color scale represented Pearson’s Correlation between the attributes of wheat across 2 cropping seasons (2022-23 and 2023-24) in response to (**a**) F70 (reduced NPK (70%)) with bacterial augmentation, (**b**) F70 (reduced NPK (70%)) regime lacking bacterial augmentation. ** represents correlation is significant at the 0.01 level (2-tailed), while * represents correlation is significant at the 0.05 level (2-tailed). **SFW**, Shoot fresh weight; **RFW**, Root fresh weight; **NT**, No. of Tillers; **SL**, Shoot length; **RT**, Root length; **NFT**, No. of Fertile Tillers; **PH**, Plant height; **SpL**, Spike Length; **SpW**, Spike weight; **PDW**, Plant dry weight; **GW**, Grain weight; **TGW**, 1000-grain weight; **P**, Grain Phosphorus; **Zn**, Grain Zn; **Fe**, Grain iron; **ZUE**, Zinc use efficiency; **FUE**, Iron use efficiency

Pearson’s correlation analysis (Fig. 3) demonstrated a biologically consistent association among traits associated with plant growth and development. The vegetative-stage morphological characteristics such as shoot fresh weight (SFW) and root fresh weight (RFW) exhibit a strong positive correlation (*r* = 0.8), signifying the interdependence of shoot and root biomass. Similarly, during the yield stage, morphological traits (e.g., plant height, plant dry weight) were positively correlated with agronomic parameters (e.g., total grain weight and 1000-grain weight). These correlations underscored the systemic progression of biomass and agronomic output, which are crucial determinants of yield in wheat crop.

The correlation chart demonstrates a highly positive relationship (*r* > 0.85) between yield-related traits and grain nutrient contents (phosphorus, zinc, iron). Moreover, P, Zn, and Fe in the un-inoculated treatment exhibit strong positive correlations with spike length, indicating that enhanced grain yield is associated with greater nutrient accumulation (Fig. 3-b). These associations are more significant under bacterial inoculation with grain phosphorus content demonstrating a significant correlation (*r* > 0.82) to various yield parameters (e.g., spike length and spike weight). Micronutrients such as Zn and Fe also exhibit highly significant correlations (*r* > 0.88) with thousand grain weight (TGW), suggesting that bacterial inoculation may contribute to the enhancement of grain weight and nutrient fortification (Fig. 3-a). The correlation analysis highlights the treatment-specific relationships among the traits. The bacterial inoculation treatment demonstrated notable positive correlations among various vegetative characteristics (e.g., VSFW and VRFW) and a significant correlation between agronomic traits and nutrient contents indicating enhanced nutrient assimilation and translocation after bacterial inoculation. The association between yield characteristics and micronutrients (Zn, Fe) was stronger under bacterial inoculation, supporting the fact that nutrient bioavailability is stimulated by bacterial activity. Weaker associations were observed in the uninoculated treatment for instance, the correlation between 1000-grain weight and spike weight are insignificant (*r* = 0.27), suggesting that the bacterial inoculation can stabilize trait relationships that influence yield stability. Moreover, the analysis demonstrates that Zn use efficiency (ZUE) and Fe use efficiency (FUE) positively correlate (*r* > 0.85) with growth parameters, yield, and grain nutrient content under bacterial inoculation (Fig. 3-a). Conversely, the uninoculated treatment illustrated the selective correlations (*r* > 0.7) of ZUE and FUE with yield traits and grain nutrient content, thereby indicating a less integrated efficiency response (Fig. 3-b).

### Genome-wide analysis

#### Phylogenetic analysis of *TaDMAS*, *TaNAAT*,* TaNAS*, and *TaVIT*

The phylogenetic tree elucidates the relationship of the wheat *TaDMAS* gene family with its relative species, primarily *Oryza sativa*, *Hordeum*

**Fig. 4:**
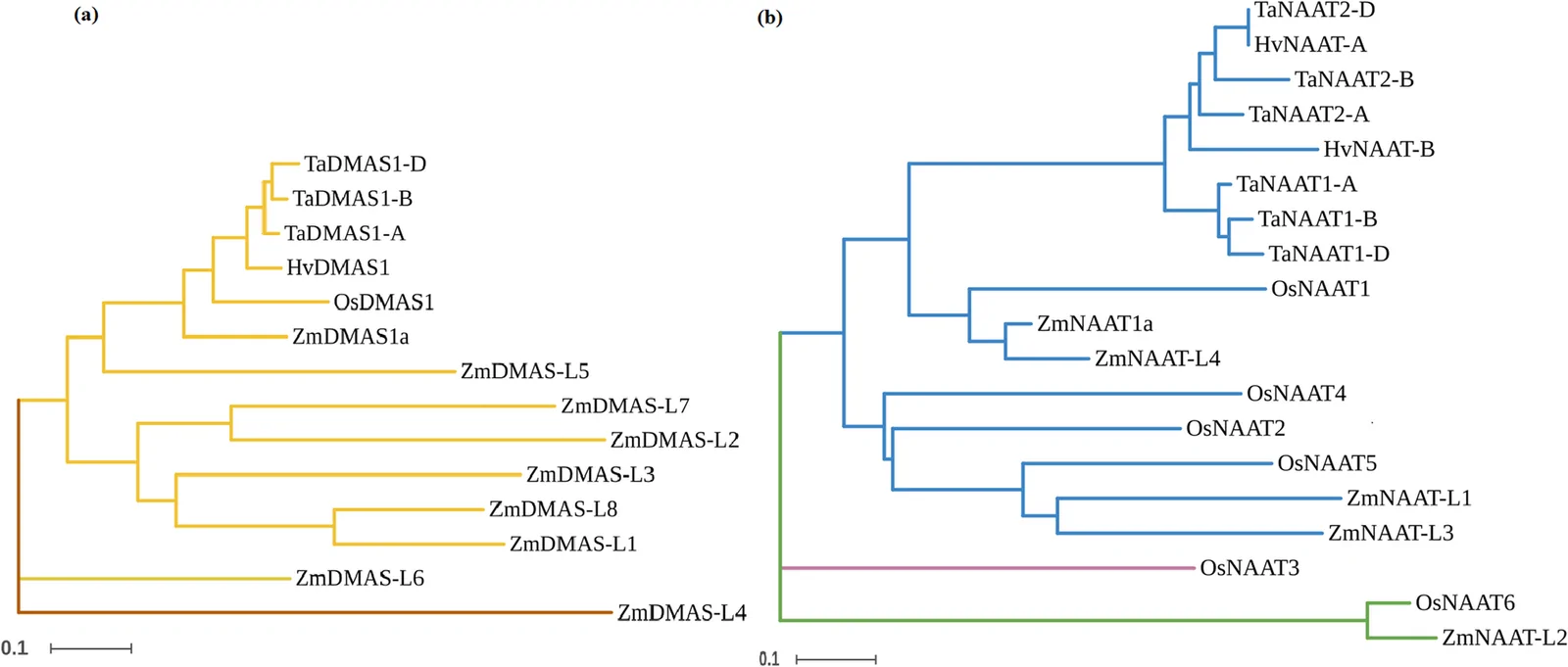
Phylogenetic analysis of (**a**) *TaDMAS* (Deoxymugienic acid synthase) and (**b**) *TaNAAT* (Nicotianamine aminotransferase) by MEGA 11 using the neighbor joining (NJ) method and further differentiated using the iTol tool

* vulgare*, and *Zea mays*. Sequences of these genes were obtained and integrated from the NCBI and Ensemble Plants database. Three *DMAS* genes were identified in wheat. A phylogenetic tree based on the neighbor-joining (NJ) method was constructed, revealing that *TaDMAS1* genes are most closely related to barley (*HvDMAS1*), followed by rice (*OsDMAS1*) and maize (*ZmDMAS1*), indicating a shared evolutionary lineage (Fig. 4-a**)**. The bootstrap consensus tree demonstrated that *TaDMAS* genes exhibit significant similarity to those of the related species, suggesting the conservation of gene function. For instance, *TaDMAS1* is positioned closer to *HvDMAS1*, implying that the functions of these proteins are conserved for the synthesis of deoxymugineic acid (DMA), which is involved in zinc and iron chelation and transport.

The positive correlation values of the analyzed wheat *DMAS* genes with barley indicate that these genes have remained syntenic and perhaps, functional across these species. This conservation is crucial for the role of *DMAS* genes in the biosynthesis of phytosiderophores, which facilitate plants in chelate micronutrients such as zinc and iron. Within the analyzed gene set, the extent of evolutionary conservation suggests that *DMAS* adopted while maintaining their functions including micronutrient regulation.

The phylogenetic analysis of the *TaNAAT* gene family revealed six *NAAT* genes in wheat (Fig. 4-b**).** These genes were compared with the homologous genes in rice, barley, and maize. The evolutionary tree demonstrated three major clades within the *NAAT* gene family, suggesting the occurrence of gene duplication events.

It was ascertained that the identified *NAAT* genes shared considerable homology with the cereal crop species, revealing evolutionary conservation. For instance, *TaNAAT1* closely grouped with *HvNAAT-B* and *OsNAAT1*, whereas *TaNAAT2* showed relatedness to *HvNAAT-A*. The presence of distant species in the phylogenetic tree clearly indicates that one NAAT gene family diversified and evolved into other functional families. This implies that wheat has undergone duplication during its evolution, as evidenced by divergent clades in the *NAAT* phylogenetic tree, which facilitate the plant’s adaptation to various environmental stressors through optimization of iron and zinc chelation and transport mechanisms. These diversified configurations within *NAAT* gene family experienced functional shifts in response to different selective pressures. Additionally, homeologs *TaNAAT1* and *TaNAAT2* were closely related to *HvNAAT-B* and *HvNAAT-A*., respectively, suggesting the functional similarity among these genes.

**Fig. 5:**
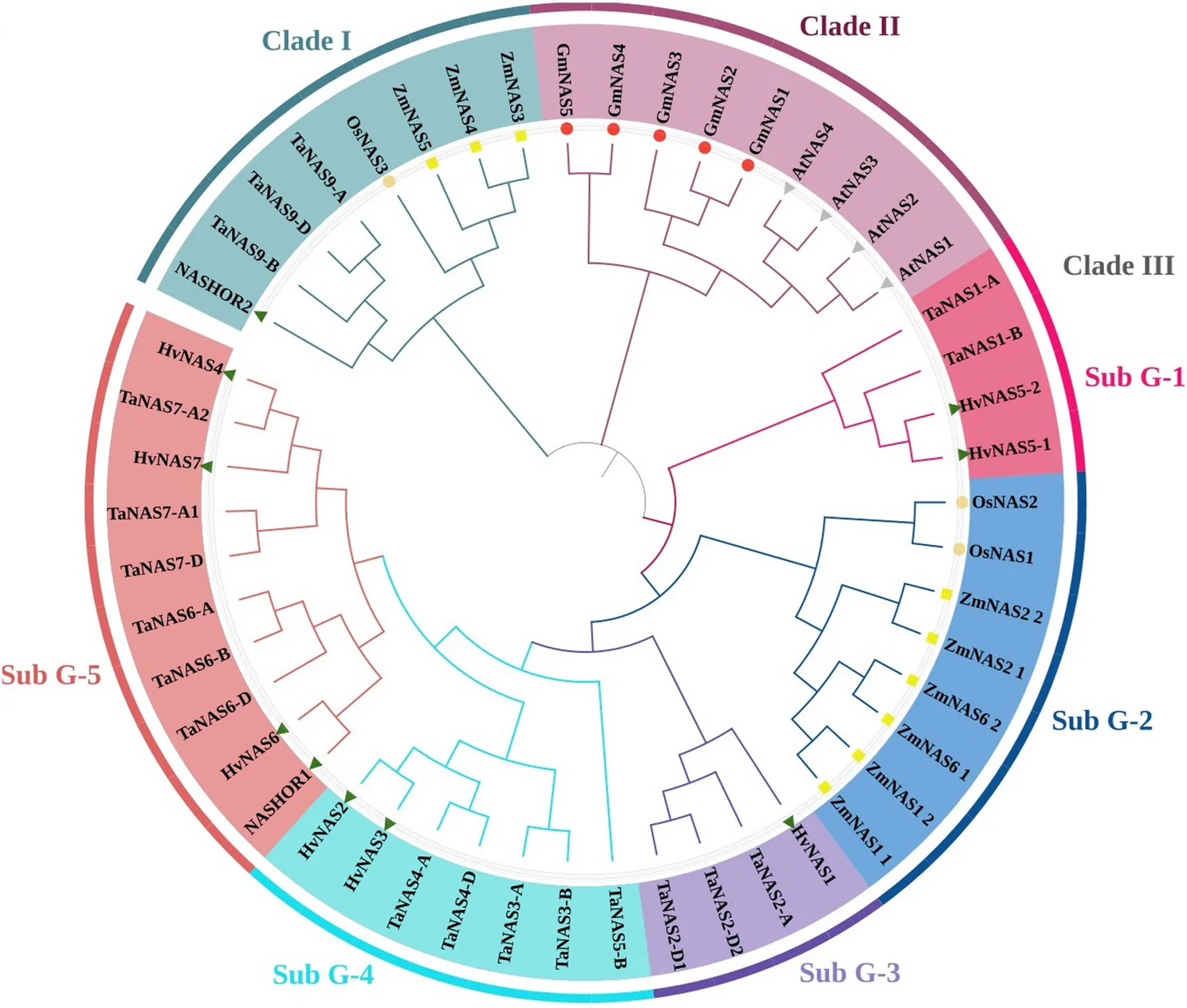
Phylogenetic analysis of *NAS* (Nicotianamine synthase) based on the protein sequences by the neighbor-joining (NJ) method using MEGA 11. Different colors illustrate the three different clades and their further subgroups. The proteins of rice, maize, barley, soybean, and Arabidopsis are indicated by different shapes

The phylogenetic analysis of *NAS* genes included gene families from rice (*OsNAS*), barley (*HvNAS*), maize (*ZmNAS*), soybean (*GmNAS*), and *Arabidopsis* (*AtNAS*) gene families. The analysis identified three distinct clades of *NAS* genes that shared significant sequence homology among the proteins. Clade I comprised 3 *TaNAS* genes, Clade II included *NAS* genes from soyabean and *Arabidopsis*, and Clade III consisted of 16 *TaNAS* genes as well as *NAS* genes from barley, rice, and maize, which were further subdivided into 5 subgroups based on the bootstrap support as illustrated in Fig. 5. The phylogenetic tree indicated that wheat *NAS* genes were more similar to *HvNAS*, suggesting an evolutionary conservation between wheat and barley NAS genes. Clade I contained 3 homeologs *TaNAS* genes (*TaNAS9*-*A*, *TaNAS9-B*, *TaNAS9-D*), which were closely related to the barley *NASHOR2* gene. Within, Clade III subgroup 1 comprises two *TaNAS* genes (*TaNAS1-A* and *TaNAS1-B*) which are most closely related to *HvNAS5-1* and *HvNAS5-2* from barley. Subgroup 2 included three *TaNAS* genes (*TaNAS2-A*, *TaNAS2-D1*, *TaNAS2-D2*) closely associated with *HvNAS1*. Subgroup 3 contained 5 *TaNAS* genes (*TaNAS3-A*, *TaNAS3-B*, *TaNAS4-A*, *TaNAS4-D*, *TaNAS5-B*), showing high similarity to *HvNAS2* and *HvNAS3*. Subgroup 5 contains 6 *TaNAS* genes (*TaNAS6* and *TaNAS7*) with 4 orthologous barley genes. The NAS gene family in wheat has undergone considerable evolutionary diversification, with the emergence of a subdivision within Clade III that further refines the functional specificity of its members, as depicted in Fig. 5.

**Fig. 6:**
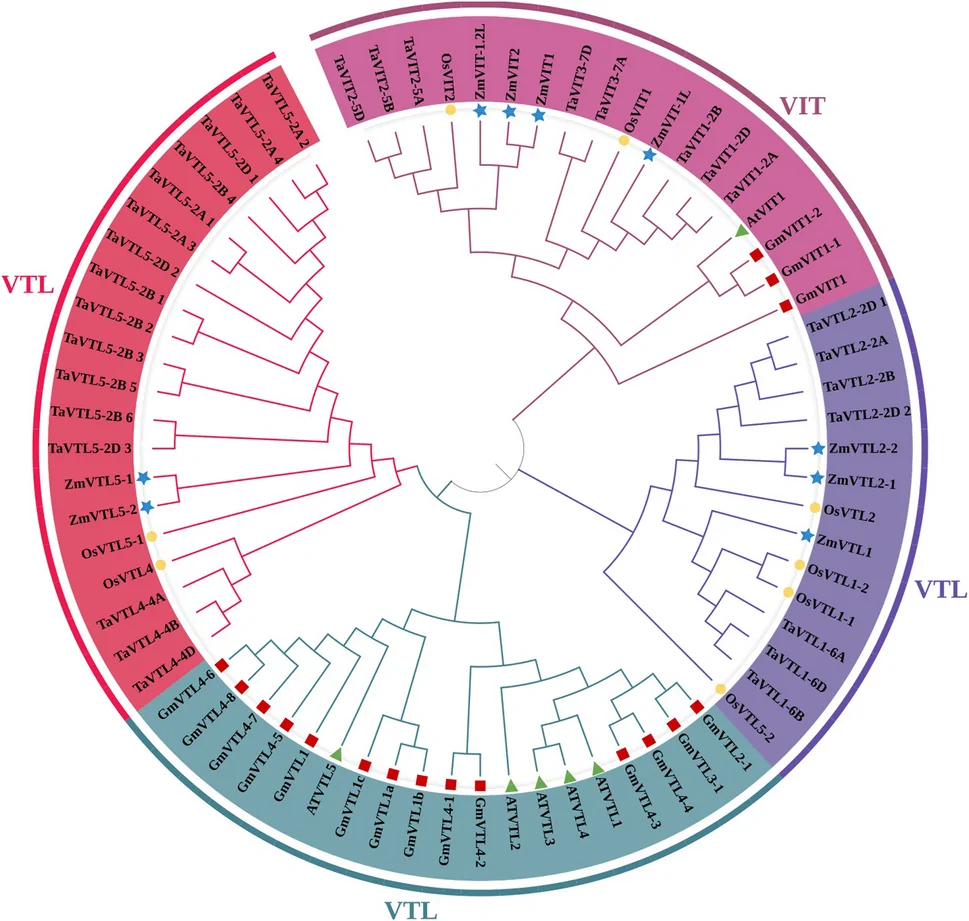
Phylogenetic analysis of *VIT* (Vacuolar iron transporter) and *VTL* (Vacuolar iron transporter-like proteins) based on the protein sequences by the neighbor-joining (NJ) method using MEGA 11. Different colors illustrate the two different clades (VIT and VTL). The proteins of rice, maize, soybean, and Arabidopsis are indicated by different shapes

The phylogenetic analysis of *VIT* and *VTL* protein sequences from wheat, rice, maize, soybean, and Arabidopsis categorized the sequences into two distinct clades: vacuolar iron transporter (*VIT*) and vacuolar iron transporter-like (VTL). The *VIT* gene family in wheat comprises 31 genes which are further subdivided into 8 *VIT* and 23 *VTL* sequences. The analysis of the *VIT* clade revealed that *TaVIT1* and *TaVIT2* each comprise of 3 homeologs, which exhibit high similarity to *OsVIT1* and *OsVIT2*, respectively. Conversely, *TaVIT3* contains 2 homeologs closely related to *OsVIT1* and *ZmVIT-1L*. The *VTL* clade comprises of 4 homeologs of *TaVTL2*, which demonstrated high similarity to *ZmVTL2* and *OsVTL2*. Additionally, both *TaVTL1* and *TaVTL4* include 3 homeologs that show high similarity to *OsVTL1*, *ZmVTL1* and *OsVTL4*. Furthermore, phylogenetic analysis identified a group of 13 highly similar *TaVTL5* sequences that clustered with high confidence alongside *ZmVTL5 and OsVTL5*, as illustrated in Fig. 6.

Two major clades of *VIT* and *VTL* genes indicate that functional differentiation occurred within the gene family. The VIT genes are involved in cellular sequestration of iron (Fe) and manganese (Mn), thereby facilitating their transport to the seed vacuole. Conversely, the role of *VTL* genes seems to be distinct, potentially involving the transport and accumulation of metals, predominantly cations. The extensive presence of *VIT* and *VTL* genes in wheat clearly indicates that this family of micronutrient transporters is primarily responsible for micronutrient storage and serve as essential regulator of micronutrient homeostasis.

#### Promoter/Cis-regulatory elemental analyses

**Fig. 7:**
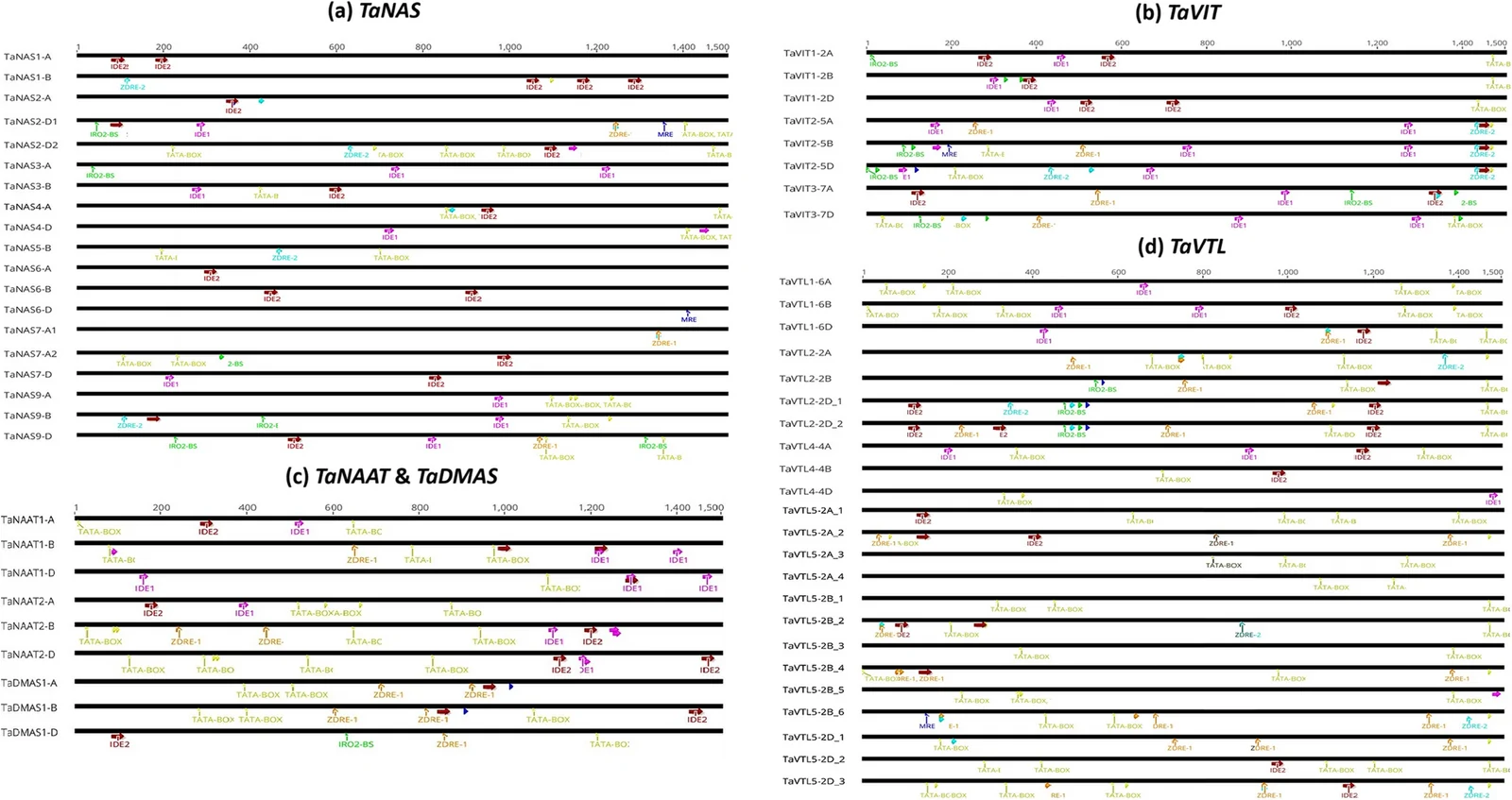
Promoter analysis of (**a**) 19 *TaNAS*, (**b**) 8 *TaVIT*, (**c**) 6 *TaNAAT* & 3 *TaDMAS*, (**d**) 23 *TaVTL* genes using Geneious Prime. The annotated cis-acting elements are illustrated as TATA-Box (TATA), Iron deficiency responsive element 1 and 2 (IDE1 and IDE2), Zinc deficiency responsive element 1 and 2 (ZDRE-1 and ZDRE-2), Iron-related transcription factor -binding sequence (IRO2-BS), and Metal-responsive elements (MRE)

The promoter study (shown in Fig. 7**)**, revealed a detailed regulatory system involving *TaNAS*, *TaNAAT*, *TaDMAS*, and *TaVIT* in response to iron and zinc deficiency. Most of the genes examined, including 3 *TaDMAS*, *TaNAAT1-B*, *TaNAAT2-B*, 11 *TaNAS*, and the majority of *TaVIT* and *TaVTL*, as depicted in (Fig. 7a-d**)**, containing *ZDRE* elements, which interact with zinc deficiency-inducible binding proteins thereby facilitating the transcription of genes under zinc-deficient condition.

Similarly, the cis-regulatory elements *IDE1* and *IDE2* were predominant within the promoter regions of *TaDMAS*, *TaNAAT*, *TaDMAS*, and *TaVIT* genes, indicating the active involvement of these promoters in response to iron deficiency. These elements are known to be bound by transcription factors associated with iron deficiency response, facilitating the regulated expression of genes crucial for iron acquisition and distribution. The identification of target sequences, as illustrated in Fig. 7 namely the *IRO2* binding sites in *TaDMAS1-D*, 6 *TaNAS*, 12 *TaVIT*, and 5 *TaVTL* genes and sequences analogous to *MRE*-like elements in *TaDMAS1-A*, *TaDMAS1-B*, *TaNAS2-D2*, *TaNA6-D*, *TaVIT2*, *TaVTL2* and *TaVTL5* confirms the participation of these genes in iron acquisition under Fe deficiency and in the regulation of other metal ions. Promoter regions containing *IRO2*-binding sequences are implicated in enhanced iron uptake, whereas the *MRE*-like elements responsive to various metal ions, suggest a broader role in metal ion regulation. The promoter regions of studied gene families host numerous *TATA-Box* elements with variable positioning, indicating the intensive transcription. The promoter analysis enhanced the understanding of the regulatory mechanisms controlling the expression of *TaNAS*, *TaNAAT*, *TaDMAS*, and *TaVIT* genes, as shown in Table 5. It also affirms their roles in micronutrient metabolism and their genetic capacity to regulate nutrient fortification in wheat.

**Table 5:** Comparative distribution of promoter elements in *TaNAAT*, *TaDMAS*, *TaNAS*, *TaVIT*, and *TaVTL* gene families

Gene families	Genes	Promoter						
		TATA-Box	IDE1	IDE2	IRO2-BS	MRE	ZDRE-1	ZDRE-2
TaNAAT & TaDMAS	6 & 3	27	13	12	1	2	8	2
TaNAS	19	26	11	17	6	3	3	8
TaVIT	8	12	13	10	12	2	12	7
TaVTL	23	70	8	18	5	4	24	13

#### Interaction analysis using string database predicting the regulatory interaction network of genes

**Fig. 8:**
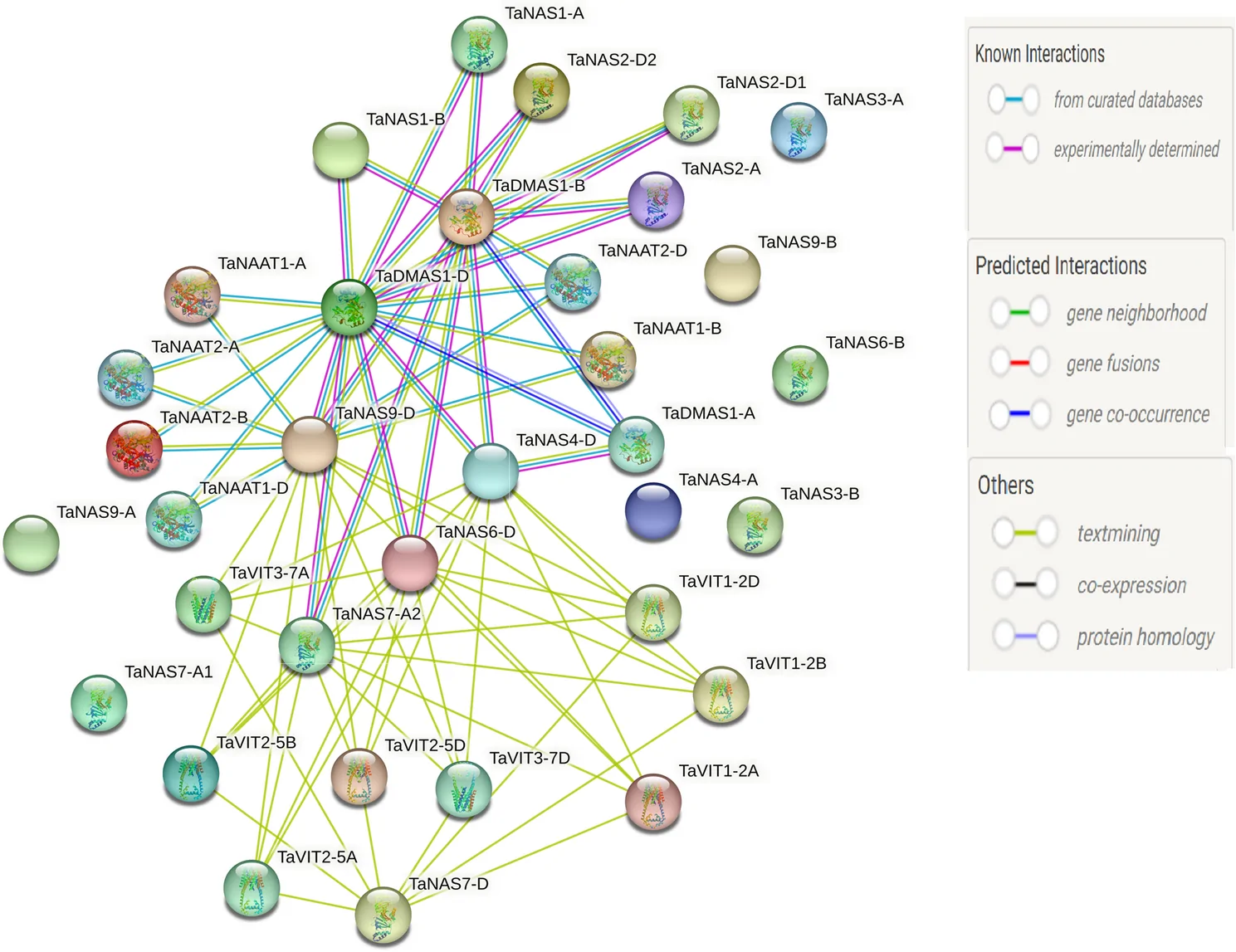
STRING network view uncovering the protein-protein interaction of complement pathways of *DMAS*, *NAAT*, *NAS*, and *VIT* genes of *Triticum aestivum* using STRING version 11.0. The string analysis was performed to gain insight into the known and predicted interactions of proteins based on reported literature. The nodes in the network represent proteins, and edges show the predicted functional interaction. Distinct colored lines depict the different types of hints used to anticipate the association. *TaDMAS*: Deoxymugineic acid; *TaNAAT*: Nicotianamine aminotransferase; *TaNAS*: Nicotianamine synthase; *TaVIT*: Vacuolar iron transporter

The behavior of genes studied through a predicted regulatory network (Fig. 8) showed interactions among phytosiderophore biosynthesis pathway proteins and Vacuolar iron transporter proteins of wheat, based on known or predicted associations, interpretations, and expression patterns. The protein-protein interaction (PPI) network obtained at a p-value: <1.0e-1 indicates weak/marginal interactions among proteins rather than strong statistical significance. However, presence of 34 nodes and 74 edges, with an average local clustering coefficient of 0.192, suggest possible functional association among the studied proteins. The proteins in the cluster belong to four gene families. *TaDMAS* (GO:0019290, GO:1990641) mainly comprises of siderophore biosynthesis and iron starvation-responsive genes, while *TaNAAT* (CL:86049) is involved in the methionine metabolism pathway and amino acid biosynthesis particularly nicotianamine aminotransferase genes. *TaNAS* (CL:175779, CL:120660, CL:120680) plays a vital role in nicotianamine and siderophore biosynthesis and also regulate as iron starvation-responsive genes. Lastly, *TaVIT* (CL:116792, CL:116790) is involved in Fe ion mitochondrial sequestration and comprises of CCC1 domian. Overall, the predicted protein interaction shows that these proteins are biologically linked as a group.

#### Protein-protein interaction pathway analysis

**Fig. 9:**
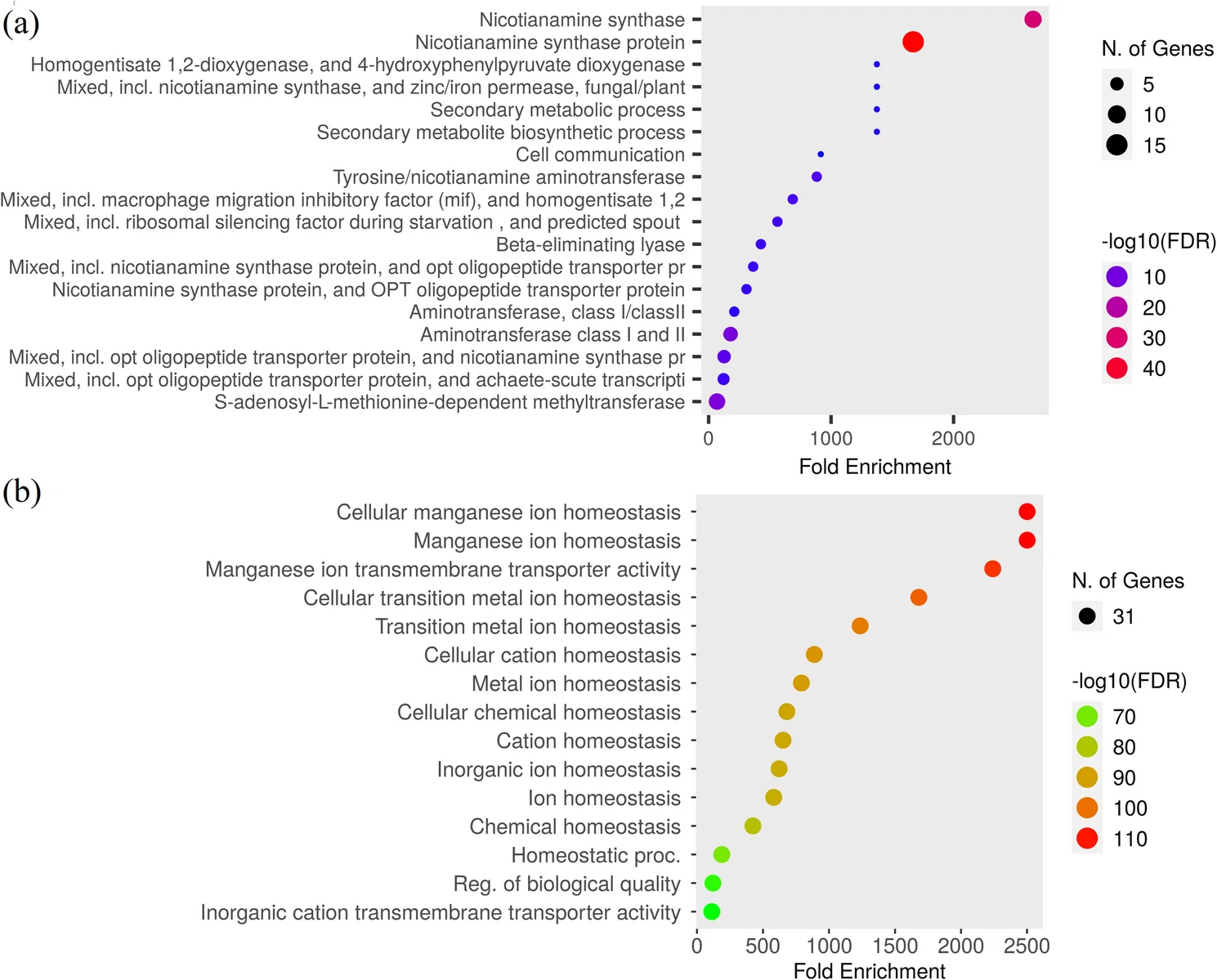
The above dot plot shows the GO pathway analysis of *TaVIT*, *TaNAS*, *TaNAAT*, and *TaDMAS* genes performed in Shiny GO vo.8 using the orthologous gene list. Each panel depicts the enriched biological processes visualized as dot plots. Each dot signifies a biological process (**a**) the top 18 most related biological processes are shown for DMAS, NAAT, and NAS, and (**b**) the top 15 predominant biological processes are shown for *TaVIT*. Each color code represents the adjusted p-value, and the size of the dot represents the number of genes

The gene ontology dot plot pathway analysis provides a comprehensive understanding of biological processes associated with 31 genes of *TaVIT*, 19 genes of *TaNAS*, 6 genes of *TaNAAT*, and 3 genes of *TaDMAS*. The predicted dot plot pathway analysis for *DMAS*, *NAAT*, and *NAS* indicated that the genes are categorized into three primary groups based on their biological processes **(**Fig. 9a**)**. One group is associated with the regulation of tyrosine and nicotianamine aminotransferase, macrophage migration inhibitory factor, and Beta-eliminating lyase. The second one includes biological processes related to the regulation of nicotianamine synthase protein, oligopeptide transporter protein, zinc or iron permease, and ribosomal silencing factor during starvation. The third group pertains to the biosynthesis of secondary metabolites (siderophores) and cell communication. In the Gene Ontology (GO) analysis for *VIT* (Fig. 9b**)**, the biological processes identified include positive regulation of cellular manganese ion homeostasis and transporter activity, transition metal ion homeostasis, cellular cation and chemical homeostasis, and inorganic cation transmembrane transporter activity.

#### Gene structure and domain analysis of *TaDMAS*, *TaNAAT*, *TaNAS*, *TaVIT* genes

**Fig. 10:**
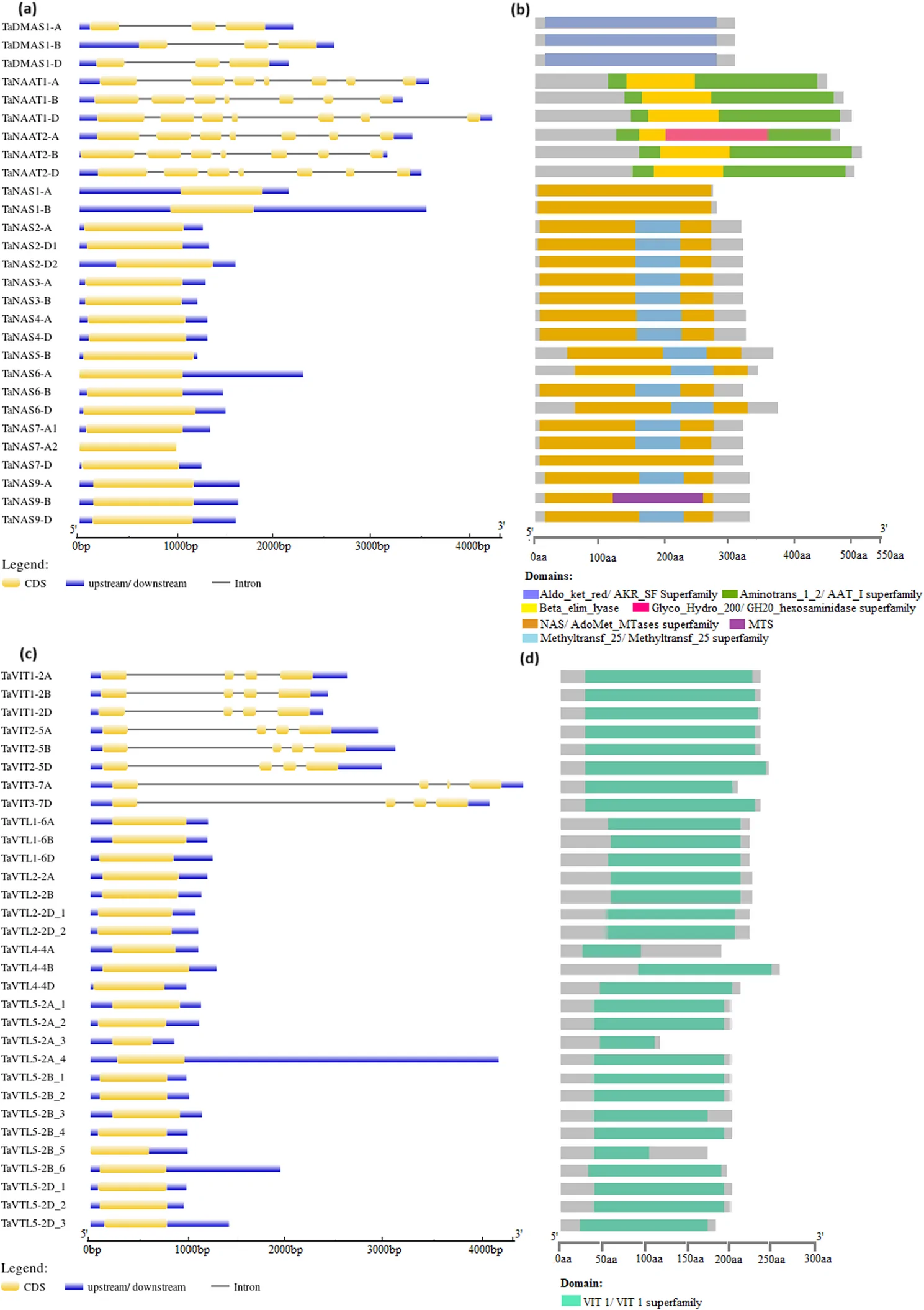
Genomic structure (left) and Domain analysis (right) were identified using gene structure display server (GSDS) and conserved domain database (CDD-NCBI), and further illustrated using tb tool (**a**, **b**) NAS, NAAT, and *DMAS* (**c**, **d**) *VIT*. Exons and introns were represented by yellow boxes and grey lines, respectively. Different-colored boxes represent different conserved domains. The scales represent the size of the genomic region (left) and the size of the protein sequence (right)

For a detailed understanding of gene structure, we performed GSDS analysis of *TaDMAS*, *TaNAAT*, *TaNAS*, and *TaVIT*. *TaDMAS* gene size ranged from 2150 bp (*TaDMAS1-D*) to 2614 bp (*TaDMAS1-B*) with three exons and two introns. *TaNAAT* gene sizes ranged from 3154 bp (*TaNAAT2-B*) to 4235 bp (*TaNAAT1-D*). The *TaNAAT* genes showed a similar gene structure with seven exons and six introns. *TaNAS* gene sizes ranged from 993 bp to 3555 bp, with *TaNAS7-A2* being the shortest and *TaNAS1-B* the longest. All *TaNAS* genes have one exonic region, with CDS lengths varying from 843 bp (*TaNAS1-A*) to 1155 bp (*TaNAS6-D*) (Fig. 10a**)**.

Based on gene structure analysis, the *VIT* gene family was classified into two subfamilies. *VIT* exhibit a similar distribution pattern concerning the number of exons and introns, whereas *VTL* consist of a single exon with no introns, as illustrated in Fig. 10c. The size of *TaVIT* genes ranged from 2173base pairs (bp) to 4034 bp, with *TaVIT-2D* being the shortest, and *TaVIT3-7 A* the longest. Conversely, the size of *VTL* gene extends from 778 bp to 3806 bp within the homoeologs of *TaVTL5-2 A*. The coding sequences (CDS) length for *VIT* varies from 557 bp to 747 bp, while for *VTL*, the CDS region mainly ranges from 549 bp to 810 bp, except for *TaVTL5-2A-3* which has the shortest size of 378 bp.

All *TaDMAS* genes were identified to contain Aldo/keto reductase superfamily conserved domain (PF00248, IPR018170) along with the NADP-dependent oxidoreductase domain (IPR023210) **(**Fig. 10b**)**. These domains suggest a role in the biosynthesis of cellular nitrogen compounds and siderophores (mugineic acid), thereby facilitating iron acquisition. The *TaNAAT* genes exhibit similar domians, including the aminotransferase, class I and II superfamily (IPR004839) and the Beta-eliminating lyase (PF01212) domain, with the exception of the *TaNAAT2-A* gene, which additionally contains GH20-hexosaminidase superfamily domain (pfam00728) as shown in **(**Fig. 10b**)**. The former domain indicates *TaNAAT’*s involvement in nicotianamine aminotransferase biosynthesis and the metabolism of carboxylic acids and organonitrogen compounds. The latter domain is associated with catalytic activity, specifically hydrolysis of glycosidic bonds. For *TaNAS*, the predominant domain was Nicotianamine synthase (PF03059), followed by the methyltransferase_25 superfamily (IPR029063) reported in 15 genes, and the *TaNAS9-B* gene uniquely containing the MTS domain. Moreover, The domain analyses suggest their role in Nicotianamine and siderophore biosynthesis as well as transcriptions factor ILR3-like response, which includes positive regulation of Fe homeostasis and plays a significant role in plant response under Fe starvation conditions. Furthermore, all *VIT* and *VTL* genes have a typical *VIT-1* or *CCC1*- family (Ca2+-Sensitive Cross-complementer) (IPR008217) domain, as shown in Fig. 10d. The domain is mainly involved in maintaining cellular iron and manganese ion homeostasis and facilitating transport to vacuoles or mitochondria. The *VTL* genes are predominantly engaged in zinc ion transport.

#### Protein motif analysis of *TaDMAS*, *TaNAAT*, *TaNAS*, and *TaVIT* genes reveals the presence of conserved domains

**Fig. 11:**
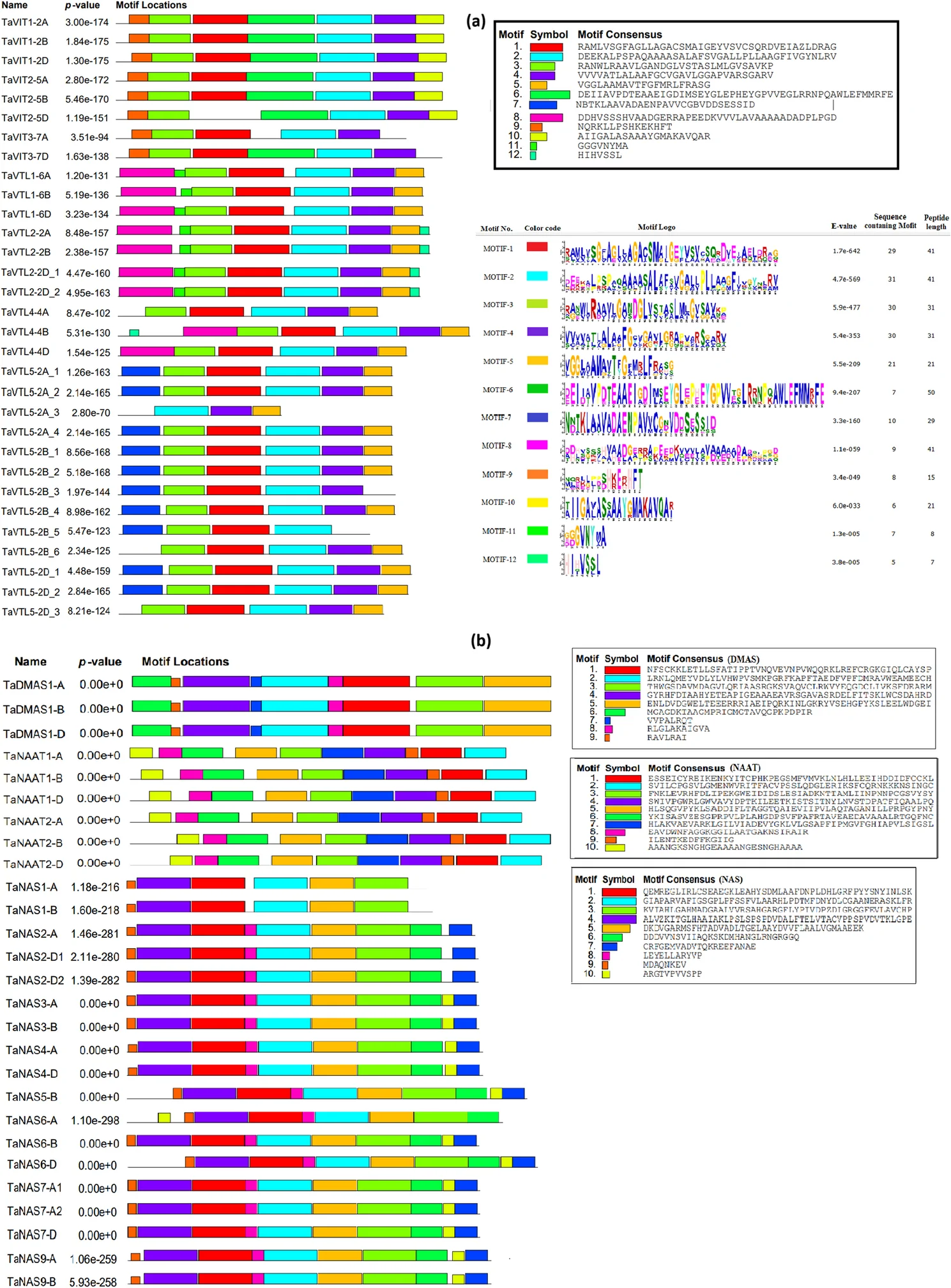
Conserved motifs identified for *TaDMAS*, *TaNAAT*, *TaNAS*, and *TaVIT* using online software MEME suite 5.5.5. The colored rectangle on each protein sequence (Black line) represents specific conserved motifs: (**a**) 12 for *TaVIT* and (**b**) 9 for *DMAS*,10 for *NAAT* and *NAS*, as shown by distinct color code. Motif consensus provides details about the precise peptide sequence and length of the conserved motif

Using the MEME tool, motif analysis was performed for *TaDMAS*, *TaNAAT*, *TaNAS*, and *TaVIT*. The motif analysis for *DMAS* illustrated the presence of 9 motifs in *DMAS*, with motifs 1, 2, 3, 8 and 9 being completely conserved throughout *DMAS* and are associated with the aldo/keto reductase domain involved in oxidoreductase activity. The motif analysis for *TaNAAT* identified 10 conserved motifs of which seven motifs (Motifs 1–7) possessed functional domains involved in pyridoxal phosphate binding (related to methionine metabolism) and the tyrosine catabolic process. Additionally, the motif analysis for *TaNAS* identified 5 conserved motifs across all *NAS* genes. Motif 6,7,8 and 9 were present in all *NAS* proteins, with certain exceptions such as motif 6 and 8 were absent in TaNAS1; motif 7 was absent in TaNAS1 and *TaNAS6-A*; motif 10 was absent in *TaNAS1* and *TaNAS2*, and motif 9 was absent in *TaNAS9-D*. Predicted motif 1–5 in *TaNAS* possess similar functional roles related to nicotianamine biosynthesis, which serves as a precursor for plant siderophore production (Fig. 11-b).

Motif analysis performed using the MEME tool identified 12 conserved motifs within wheat *VIT* proteins. All *TaVIT* proteins possess motifs 1 to 4, 6, 9, 10, except for 3 genes that lack certain motifs TaVIT2-5D, *TaVIT3-7A* and *TaVIT3-7D*. Motifs 9 and 10 were specific to *TaVIT*, with the exception that motif 10 is absent in *TaVIT3-7A*. Similarly, motifs 1 to 5, 7, 8,11, and12 were present in *VTL* genes, with motif 5 present in all *TaVTL* genes, and other motifs being specific to certain genes including motif 8 to *TaVTL*1,2 and 4; motif 11 to *TaVTL*1, 2 and *TaVTL4-4B*, motif 12 to *TaVTL2*, and Motif 7 to *TaVTL5*, except for *TaVTL5-2 A-3*, *TaVTL5-2B-6* and *TaVTL6-2D-3*. All motifs within *VIT* and *VTL* proteins are associated with the CCC1 family domain, which is primarily involved in the cellular sequestration of iron, manganese, and zinc ions in plants (Fig. 11-a).

#### *DMAS*, *NAAT*, *NAS*, *VIT*, and *VTL* gene expression pattern in different wheat developmental tissues

**Fig. 12:**
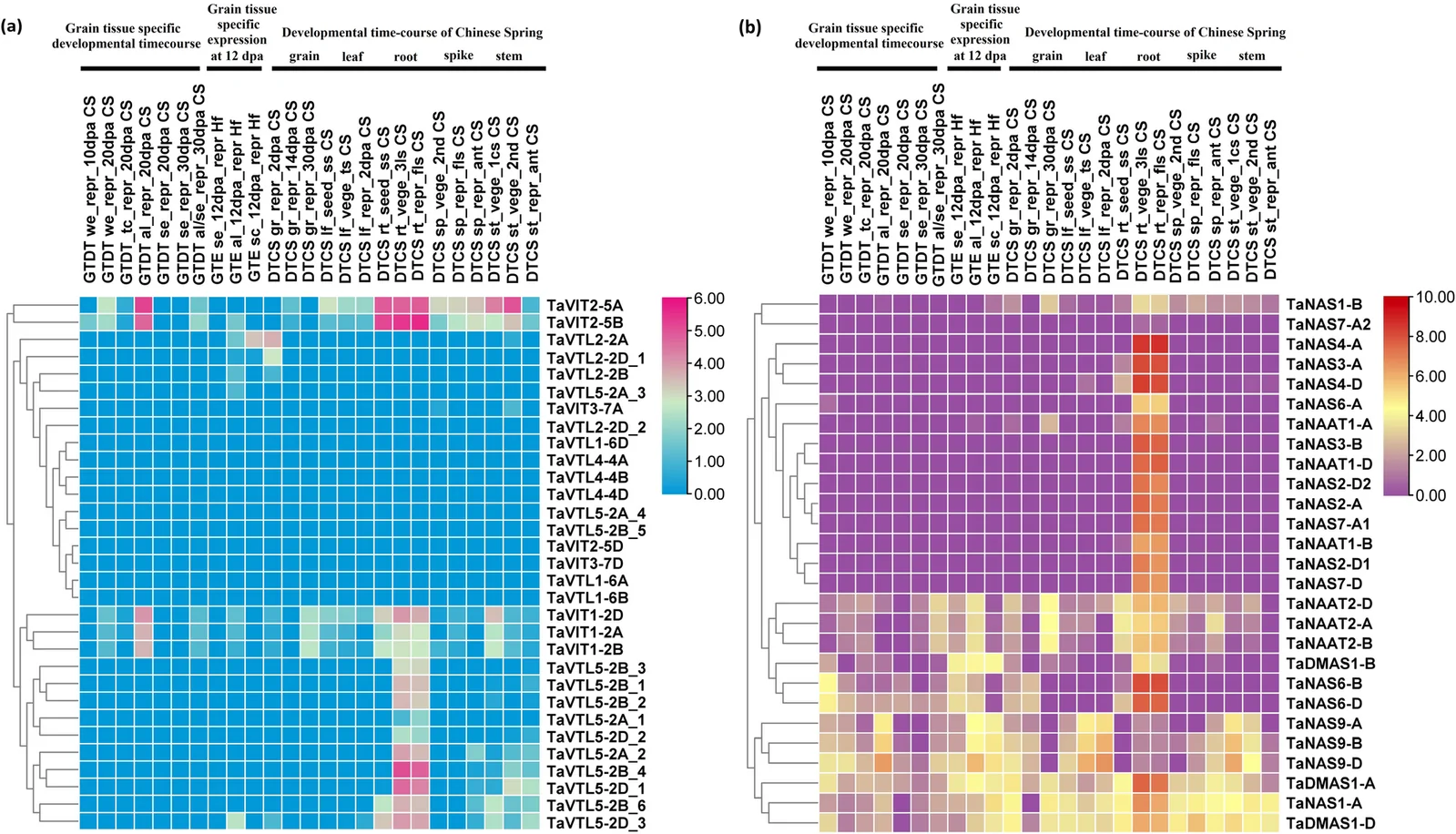
The heatmap illustrates the relative expression profile of putative **(a)** *TaVIT*, (**b**) TaNAS, TaNAAT, and TaDMAS generated using expression values in transcript per million (TPM) from the expVip database. Change in the color bar from blue to pink **(11a)** and purple to red **(11b)** depicts an increase in transcript expression. The heatmap shows the expression of different wheat tissues (root-rt, stem-st, spike-sp, leaf-lf, grain-gr, whole endosperm-we, aleurone-al, starchy endosperm-se, seed coat-sc) at various time points and developmental stages, including seedling, vegetative, flag leaf, tillering, and 10,12,20,30 days post anthesis (dpa)

The expression patterns of *VIT* and *VTL*, which are involved in the transport and regulation of iron, as well as *DMA*S, *NAAT*, and *NAS*, crucial for the biosynthesis of nicotianamine and deoxymugineic acid in wheat (*Triticum aestivum*), were studied. Among the *TaVIT* gene family, transcripts of *TaVIT2-5 A*, *TaVIT2-5B*, and *TaVIT1* exhibited high levels of expression in root and grain tissues, particularly during the later stages of grain development (GTE and GTDT). Conversely, *TaVTL5* demonstrated higher transcript levels in root tissues. The majority of *TaVIT* genes were undetectable in most tissues, indicating low expression levels. Notably, *TaVIT2-5A* and *TaVIT2-5B* showed enhnaced expression during grain development (GTDT-20 dpa), with low expression observed in grain and leaf tissues during early developmental stages. This pattern suggests that *TaVIT2* genes play a significant role in the storage and transportation of iron during grain filling, facilitating iron accumulation as the grain matures (Fig. 12a).

The expression profile of *TaNAS*, *TaNAAT*, and *TaDMAS* genes revealed two primary patterns across different tissues. Notably, the transcript abundance of *TaNAS*, *TaNAAT*, and *TaDMAS* was significantly higher expression in root tissues. The dominant expression of these genes in roots suggests that they play crucial roles in phytosiderophore production, which facilitates the translocation of nutrient from soil to aerial parts during early vegetative and reproductive growth. Specifically, genes *TaNAS*1, *TaNAS*6, *TaNAS*9, *TaNAAT2*, and TaDMAS1 reported considerable transcript abundance in root tissues especially in DTCS veg, with moderate expression observed in other tissues, especially in grain tissues, as well as at different developmental stages. The grain-specific expression suggests additional functions related to seed development and iron remobilization. Conversely, the remaining genes, including *TaNAS2–5*, *TaNAS7*, *TaNAS8*, and *TaNAAT2*, displayed low transcript accumulation (deep purple), predominantly in tissues other than roots, implying a low contribution in metal chelation and transport within specific tissues as depicted in Fig. 12b.

### qRT-PCR analysis of Zn and Fe transporter genes in the shoot of contrasting wheat genotypes in response to Zn and Fe-deficient conditions

The qRT-PCR analysis of two wheat genotypes (Akbar-19: Zn biofortified and Khaista-17: Low Zn cultivar) was performed to get an insight into the regulation of *NAS*, *NAAT*, *DMAS*, and *VIT* genes in the shoot under Zn and Fe stress. Compared to the control, transcript levels of the studied genes in both genotypes were elevated across all the treatments, with the most prominent upregulation observed in khaista-17 under consortia-inoculated Fe-deficient conditions. Relative to Akbar-19, Khaista-17 exhibited markedly enhanced expression approximately twofold in shoot tissue for *NAS*,* NAAT*,* DMAS*, and *VIT* under all treatment conditions.

**Fig. 13:**
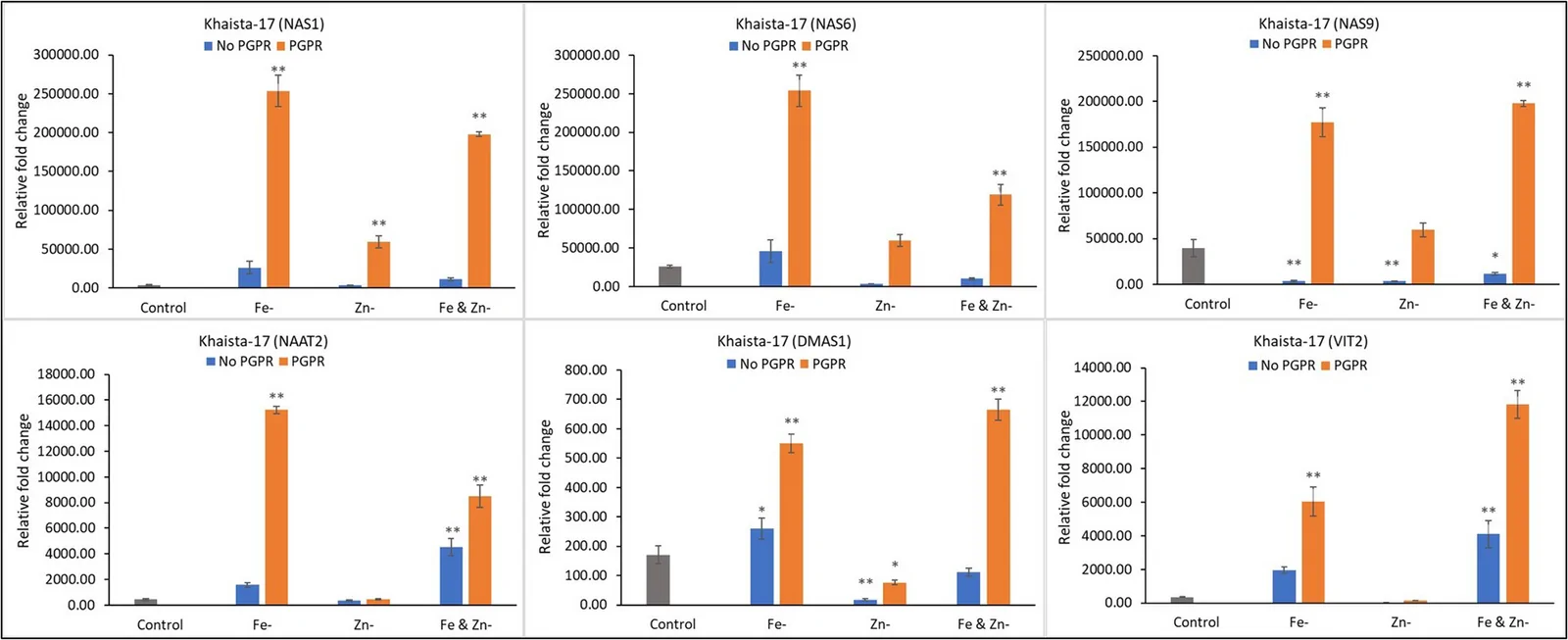
The expression analysis of phytosiderophore production gene (*NAS1*, *NAS6*, *NAS9*, *NAAT2*, *DMAS1*) and Vacuolar iron transporter gene (*VIT2*) in 4-week-old shoot of Khaista-17 (local wheat cultivar). The grey bar represents the control group, the blue bar indicates the uninoculated plants, and the orange bar shows the inoculated plants. C- Standard Hoagland solution, Fe-insoluble iron (Fe_2_O_3_), Zn- insoluble Zn (ZnO), Fe & Zn- insoluble iron (Fe_2_O_3_) & insoluble Zn (ZnO)

**Fig. 14:**
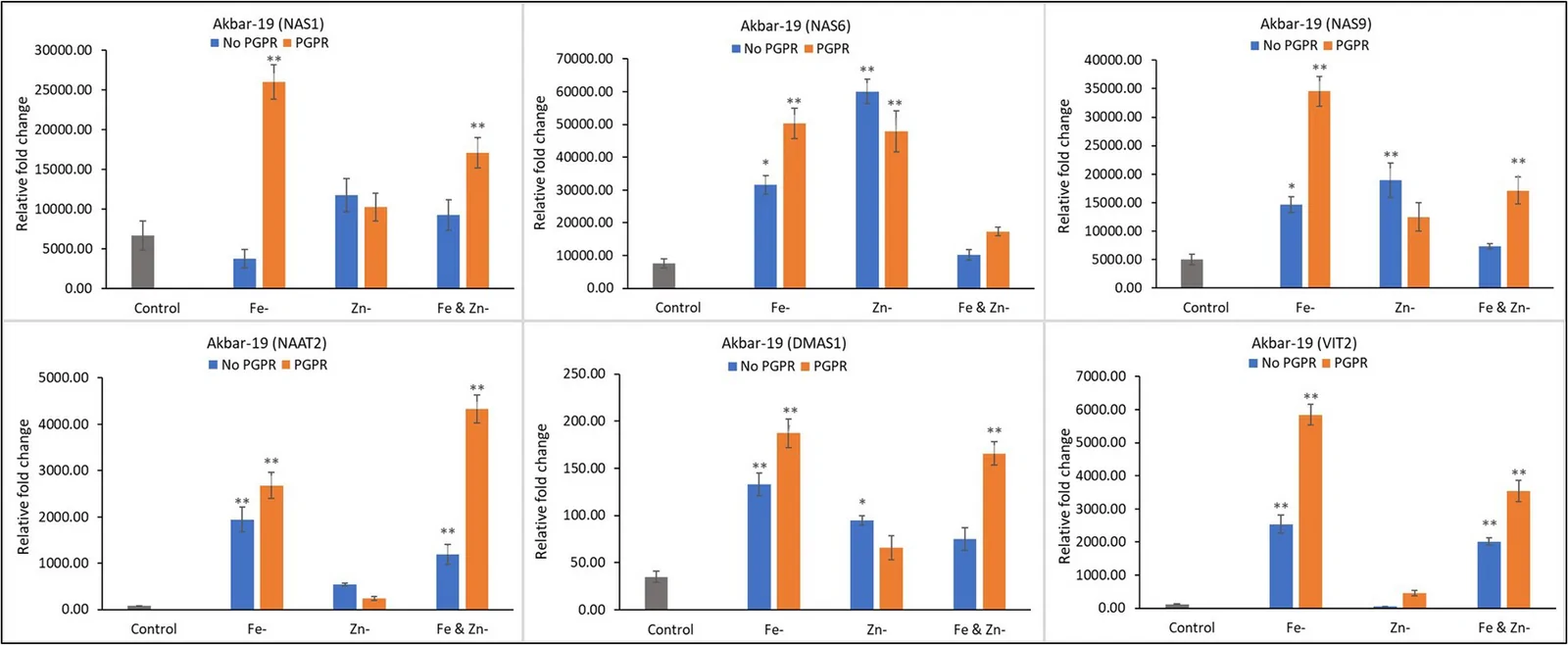
The expression analysis of phytosiderophore production gene (*NAS1*, *NAS6*, *NAS9*, *NAAT2*, *DMAS1*) and Vacuolar iron transporter gene (*VIT2*) in 4-week-old shoot of Akbar-19 (Zn-biofortified wheat cultivar). The grey bar represents the control group, the blue bar indicates the uninoculated plants, and the orange bar shows the inoculated plants. C- Standard Hoagland solution, Fe-insoluble iron (Fe_2_O_3_), Zn- insoluble Zn (ZnO), Fe & Zn- insoluble iron (Fe_2_O_3_) & insoluble Zn (ZnO)

Gene expression analysis revealed that *TaNAS1*, *TaNAS6*, and T*aNAS9* were consistently expressed in the shoot tissues across all the treatments. In Khaista-17, consortia-inoculated plants exhibited significantly higher expression of all 3 *TaNAS* genes by day 28, compared to control and uninoculated plants across all treatments, as shown in Fig. 13 (a-c). Moreover, the relative fold expression of *TaNAS1*, *TaNAS6*, and T*aNAS9* genes when treated with PGPR demonstrated highest induction relative to micronutrient stress alone, as detailed in Table 6. Similarly, Akbar-19 inoculated with consortia, showed significantly higher expression in all 3 *TaNAS* genes under both Fe-deficient and combined Zn and Fe deficient conditions. The enhanced expression level indicates an increased demand for NA-mediated Fe and Zn transport in inoculated plants. Under Zn-deficient conditions, Akbar-19 showed higher expression levels of *TaNAS1*, *TaNAS6*, and T*aNAS9* in uninoculated plants with notable fold changes of 0.8, 3, and 1.9, respectively, as shown in Table 6. This suggests that the genotype-specific response of Akbar-19 activates Zn-responsive pathways, leading to enhanced Zn uptake and transport in the absence of microbial support, as illustrated in Fig. 14 (a-c).

**Table 6:** The table presents the expression of *NAS1*, *NAS6*, *NAS9*, *NAAT1*, *DMAS1*and *VIT2* in Khaista-17 (local cultivar) and Akbar-19 (Zn-biofortified wheat variety) under Fe, Zn, combined Zn and Fe stress alone and along with PGPR treatment relative to control. Expression values are presented as Log2fold change relative to untreated control. The control was used as the calibrator and therefore assigned a log2FC value of 0, corresponding to no change. Positive values show upregulation, Negative values show the downregulation, and 0 indicates no change relative to control

Variety	Genes	Control	Fe stress	Fe stress +PGPR	Zn stress	Zn stress + PGPR	Zn & Fe stress	Zn & Fe stress + PGPR
(Khaista-17)								
	NAS1	0	3.0	6.3	0.0	4.2	1.8	5.9
	NAS6	0	0.8	3.3	-3.0	1.2	-1.3	2.2
	NAS9	0	-3.5	2.2	-3.6	0.6	-1.8	2.3
	NAAT2	0	1.8	5.1	-0.3	0.1	3.4	4.3
	DMAS1	0	0.6	1.7	-3.3	-1.2	-0.6	2.0
	VIT2	0	2.4	4.1	-3.7	-1.4	3.5	5.0
(Akbar-19)								
	NAS1	0	-0.8	2.0	0.8	0.6	0.5	1.4
	NAS6	0	2.0	2.7	3.0	2.6	0.4	1.2
	NAS9	0	1.5	2.8	1.9	1.3	0.5	1.8
	NAAT2	0	4.7	5.1	2.8	1.6	4.0	5.8
	DMAS1	0	1.9	2.4	1.4	0.9	1.1	2.2
	VIT2	0	4.4	5.6	-1.0	1.9	4.1	4.9

In both genotypes, *TaNAAT2* and *TaDMAS1* showed significantly increased expression levels in the shoot tissues under conditions of Fe-deficiency and combined Zn and Fe stress. Notably, inoculated plants demonstrated markedly higher expression levels compared to control and other treatments, as illustrated in Fig. 13 (d, e). The higher positive fold change values of *TaNAAT2* and *TaDMAS1* under inoculated conditions across both genotypes are detailed in Table 6. Under zinc deficiency, inoculated plants of Khaista-17 showed expression level comparable to those of the control and uninoculated treatment. Conversely, Akabr-19 (Zn-biofortified) exhibited enhanced expression in both inoculated and uninoculated plants relative to the control, as depicted in Fig. 14 (d, e). The presence of cis-regulatory elements IDE1, IDE2, and ZDRE1 within the promoter regions of *TaNAAT2* and *TaDMAS1* suggests their induction under conditions of Zn and Fe deficiency.

*TaVIT2* is a crucial iron transporter localized to the vacuole and represents a potential gene for facilitating iron transport into the vacuole. Under Fe deficiency and combined Zn and Fe stress, an enhanced expression of *TaVIT2* was observed in plants relative to the control, with significantly higher expression levels noted in inoculated plants across both genotypes. Conversely, under Zn deficiency, *TaVIT2* was upregulated in inoculated plants compared to the uninoculated and control groups in Akbar-19, however, in Khaista-17, increased expression was observed in inoculated plants compared to uninoculated plants, as illustrated in Figs. 13 and 14 (f).

Compared to the transcriptional response of two genotypes (refer to Table 6), Akbar-19 demonstrated a more pronounced stress-induced expression of all studied genes than Khaista-17 under Zn, Fe, and combined (Zn and Fe) deficiency conditions, particularly in the absence of bacterial consortia. This was notably linked to its superior ability for Fe/Zn chelation and vacuolar loading. The application of bacterial consortia significantly upregulated these genes in Khaista-17; however, in Akbar-19 the genes were strongly induced by nutrient stress and only moderately stimulated by PGPR, thereby suggesting improved genetic mechanism for micronutrient homeostasis in biofortified cultivar, Akbar-19.

## Discussion

### Modulation of yield and micronutrient partitioning via PGPR-mediated biofortification

Cereals, especially wheat, possess an inherently low Zn and Fe content, which limits their capacity to meet human dietary zinc requirements, especially in regions characterized by marginal soils and high prevalence of micronutrient deficiency [29]. Chemical fertilizer applications are less efficient in boosting grain micronutrient concentrations due to high pH and calcareous nature of soils that promote immobilization of these chemicals [30]. PGPR significantly improve nutrient solubilization and absorption in the soil thus contribute to enhance crop productivity [31]. The present study demonstrates the potential of PGPR under zinc and iron-limiting conditions to mobilize and translocate these micronutrients in wheat and their likely contribution to produce biofortified grains. This approach presents a sustainable strategy to improve Zn and Fe uptake, nutrient use efficiency, nutritional value, and crop yield [32, 33].

The present study showed the efficacy of designed bacterial formulation to improve productivity and micronutrient fortification, in two wheat genotypes, Akbar-19 (Zn-biofortified) and Khaista-17 (Low-Zn) under field conditions. Both genotypes demonstrated a positive response to bacterial consortia under reduced fertilizer application (F70) in both growing seasons for most of the morphological and yield related traits. Interestingly, the F70 + B + Zn/Fe treatment performed better than F100 (control), indicating the synergistic effects of bacterial inoculation combined with micronutrient supplementation on observed plant traits and nutrient content. It further emphasizes that bacterial inoculation improves the efficiency of applied fertilizer, thereby accelerating plant metabolic enzyme activities and ultimately improving yield. These results further validate that Zn-solubilizing bacteria along with chemical fertilizers enhanced Zn uptake and yield of wheat in Zn-deficient soils [12, 34, 35, 36]. The genotype response was found variable in the present study where the Akbar-19 exhibited improved plant height and spike weight compared to Khaista-17, indicating inherent genetic variability in nutrient utilization efficiency and responsiveness to biofertilization.

A significant positive correlation observed between yield components, morphological traits, and grain nutrient contents (P, Zn, Fe) shows that bacterial inoculation improved not only the growth (morphological, agronomic traits) but also the nutrient solubilization and its fortification into the edible part. Earlier studies showed that combining bacterial inoculation with supplemental inputs (e.g., foliar nano‑Zn) consistently improves nutrient uptake, stimulates growth in cereals and sugarcane, and ultimately enhances yield [37, 38].

Akbar-19 is reported as a high yielding variety demonstrating consistent stability over several years showing up to 7.05% increase than other varieties in trials at multiple locations across Pakistan [39]. Similarly, the yield performance of Khaista-17 has also been tested across various locations in Khyber Pakhtunkhwa [40]. Present study shows that both Akbar-19 and Khaista-17 exhibited improved performance under bacterial and zinc-amended reduced NPK regimes, thereby suggesting rhizobacterial-assisted enhanced nutrient use efficiency and potentially higher yields.

PGPR-mediated enhanced grain nutrient uptake (P, Zn, and Fe) across both genotypes demonstrate efficient nutrient absorption and translocation from soil to grain. Of both genotypes, Akbar-19 exhibited the maximum grain Zn and Fe (40.2 and 48.2 mg/kg, respectively). The data confirms the previous findings [41, 42, 43] which demonstrate that nutrient-solubilizing bacteria can enhance the Zn and Fe content of wheat and rice grains. It further affirms the role of bacterial inoculants in the solubilization and mobilization of soil nutrients, ultimately enhancing the nutritional quality of the crop.

The study was further extended to include genome-wide analysis to get an insight into the genetic mechanisms responsible for micronutrient fortification in wheat, along with molecular study that validates the genotype-specific nutrient transporter response across both genotypes.

### Phylogenetic and regulatory insight of strategy II micronutrient (Zn and Fe) acquisition genes

Micronutrients, mainly Zn and Fe, are important for crop physiological processes and these processes are severely affected under nutrient-deficient soil conditions, ultimately impacting crop productivity [44]. The reduced nutrient content in cereal crops, mainly wheat, is primarily due to stringent soil conditions that influence grain yield. Graminaceous plants have adopted a unique strategy to mitigate deficiencies of these vital nutrients. In Wheat, the strategy II nutrient uptake mechanism is commonly used to absorb Zn and Fe from the rhizospheric zone into the plant roots, where phytosiderophore exudation mediated by mugineic acid (MA) family proteins facilitates the chelation of these nutrients forming complexes such as Zn^2+^-MA and Fe^3+^-MA [17]. In wheat, the gene families *TaNAS*, *TaNAAT*, and *TaDMAS* are integral component of phytosiderophore biosynthesis pathway [15, 45]. Furthermore, *VIT gene* is involved in metal homeostasis and serves as promising candidate for essential micronutrient biofortification in cereal grains. Specifically, in wheat, the role of *VIT* and *VTL*-like protein from the MFS gene family are crucial for the mobilization and translocation of Zn and Fe to foliar tissues and seeds [46].

The studies have identified NAS, NAAT, DMAS and VIT gene families in wheat but, a comprehensive genome-wide analysis of their phylogenetic relationships, promoter analysis, gene interaction and pathway studies, gene structure, domain and motif characterization are yet to be reported. This gap was undertaken in the current study which elucidates the phylogenetic relationships of wheat with several relative species, including rice, barley, maize as well as Dicot/legume (soyabean), and model plant (*Arabidopsis*). Phylogenetic analysis revealed a close relationship of wheat and barley *DMAS*, *NAAT*, and *NAS* genes, indicating potential evolutionary and functional similarities between wheat and barley. Furthermore, as reported earlier [47], the phylogenetic analysis of *TaNAAT* revealed that wheat *TaNAAT1* and *TaNAAT2* genes have undergone duplication in the diploid wheat ancestor following divergence, likely adapting for roles in nutrient chelation and transport. The phylogenetic tree of the *NAS* genes family indicated the presence of multiple clades and subgroups, reflecting substantial genetic and physiological diversity within this gene family. Furthermore, the *TaNAS* gene family in wheat comprises a higher number of genes relative to other species, suggesting their extensive involvement in metal ion regulation influenced by the mineral content of the soil. This finding validates a recent study [48], which indicated that gene duplication events have contributed to the expansion of the NAS gene family in wheat, supporting the concept of subfunctionalization.

In contrast to NAS, NAAT and DMAS, the phylogenetic analysis of wheat *VIT* and *VTL* demonstrated the evolutionary divergence showing close clustering with rice and maize *VIT/VTL*. A high number of *VTL* genes in wheat is attributable to multiple homologous regions, supporting the previous findings [46] which reported the occurrence of duplication events of *VTL* genes in wheat. Notably, the highest count of *VTL* genes was observed on chromosome 2, which is linked with quantitative trait loci (QTL) associated with fortifying Zn and Fe contents in grains. This suggests the involvement of *TaVTL* in the localization of Zn and Fe within seed vacuoles; however, their role beyond metal transport and storage remains unspecified. These phylogenetic analysis provides a structural foundation for understanding the evolutionary and functional relationships between homologous protein sequences across different species.

Present study demonstrates six cis- regulatory elements to be associated with zinc and iron stress including the Iron deficiency responsive elements (IDE1 and IDE2), Zinc deficiency responsive elements (*ZDRE-1* and *ZDRE-2*), Iron-related transcription factor -binding sequence (*IRO2-BS*), and Metal-responsive elements (*MRE*). The promoter analysis (Table 5) revealed significant counts of IDE1, IDE2, ZDRE-1, and ZDRE-2 in *TaNAAT*, *TaDMAS*, and *TaNAS*, as reported earlier [49] confirming the presence of IDE1 and IDE2 in the promoters of *TaNAAT*,* TaDMAS*, and *HvNAAT.* This suggests the presence of binding sites responsive to Fe deficiency that are involved in Fe homeostasis [50]. The studies have shown the presence of ZDREs in the promoter regions of ZIP transporter genes in wheat, rice and barley, as well as the binding of specific transcription factors to these promoters under Zn deficiency, thereby confirming their role in Zn deficiency responsive gene regulation [27,51]. The presence of IDE1, MRE, and IRO2 in the promoter region of *TaVIT* has been reported earlier [46] which aligns with our study, indicating the vital role of *TaVIT* in maintaining metal ions homeostasis and modulating its gene expression in response to changes in metal ion levels, primarily Zn and Fe.

Gene structure analysis indicated that all 3 *TaDMAS* genes are positioned on chromosome 4 with nearly almost similar sequence lengths. Whereas, *TaNAAT* genes are located on chromosome 1, with a little variation in sequence length. The structural analysis of *NAS* genes reveals its distribution across all chromosomes except chromosome 1 and 7. Furthermore, *TaVIT* are situated on chromosomes 2, 4, 5 and 7 with significantly varying sequence lengths observed for both *TaNAS* and *TaVIT* genes.

The regulatory networks of *DMAS*, *NAAT*, *NAS*, and *VIT* proteins in wheat, exhibited substantial protein-protein interactions (PPI) with p-value of less than1.0e-1, indicating marginal interaction among proteins from different gene families. Protein-protein interaction prediction revealed the role of *TaNAS* in methionine metabolism and nicotianamine synthesis, as well as the role of *TaNAAT* genes in nicotianamine aminotransferase synthesis [16]. Moreover, *TaDMAS* genes are essential for siderophore synthesis (nicotianamine (NA)) and for iron regulation. The *TaVIT* genes demonstrate retained functionality similar to the CCC1 domain in yeast that is associated with vacuolar iron storage and redistribution. Moreover, VTL proteins predominantly mediate Zn sequestration into vacuoles, further reinforcing the functional coherence of this transporter set. These results are consistent with earlier transcriptomic studies [17], which reported significant regulation of these genes under Zn and Fe-deficient conditions, thereby, reinforcing the potential of these proteins as targets for phytosiderophore biosynthesis. This suggests enhanced nutrient uptake under stress conditions, facilitating metal ion sequestration within organelles, and maintaining metal homeostasis. Additional in‑depth research is needed to validate the functions of these genes and to clarify their interaction dynamics within broader regulatory pathways.

The gene expression analysis provided further insights into the tissue-specific developmental regulation of NAS, NAAT, DMAS, VIT, and VTL genes in wheat, revealing variability in gene activity across various tissues and developmental stages. A prominent expression observed in *TaNAS* genes in roots suggests that NA serves as precursor to phytosiderophore (DMA), which is released from plant roots into the rhizosphere to chelate Fe^3+^. Generally, Clade III *TaNAS* genes play key role in root-specific NA synthesis during vegetative as well as reproductive stages. On contarary, Clade I *TaNAS9* exhibits shoot-specific NA production throughout the grain filling stage and notably at 12-days post- anthesis (dpa). Similar predominant expression of *TaNAS* genes in seedling root, leaves, shoot and at reproductive stages in anther and seeds is reported earlier [48], suggesting tissue specific expression pattern.

The expression of *TaDMAS* across different tissues and grain development stages predicts its role in long-distance nutrient transport and its loading into the wheat grain. A similar trend has been reported in rice and maize *DMAS* genes based upon the phylogenetic relationships and expression profiling [52]. The expression analysis of *TaNAAT* depicted variations in regulation, where *TaNAAT2* was observed being expressed in most tissues and grain development stages, primarily the root, spike, and grains, while *TaNAAT1* exhibited root-specific expression. The results aligns with barley *HvNAAT* genes [47], which are orthologs of *TaNAAT*, both showing an enhanced expression in anther and grain tissues, indicating a significant role in anther development and reproduction. Root-specific *TaNAS*, *TaNAAT*, and *TaDMAS* genes are expressed during vegetative growth as active iron uptake genes; however, others also involved in seed development potentially through chelation and redistribution of iron to nourish the embryo and endosperm [47].

Expression profiling of *TaVIT1* and *TaVIT2* demonstrated higher transcript accumulation in the roots, shoot, and spike. During the grain filling stage, higher transcript levels were observed in the aleurone layer 20 days after anthesis (dpa) in *TaVIT2* and *TaVTL5*. It has been previously reported that *TaVIT1* can be a good candidate for enhanced absorption and accumulation of Fe and Zn in the grain [53, 54] while the endosperm specific expression of *TaVIT2* and *TaVTL5* is already established [55, 56]. The studies also explained that iron is captured from the large flux of iron to the embryo’s scutellum through the endosperm adjacent to the scutellum and was retained there 20 days post anthesis [55, 56]. The *TaVIT2* genes are linked to the grain and regulated to facilitate iron sequestration within seeds.

Overall, the expression analysis indicated major physiological events associated with specific gene families including *NAS*, *NAAT*, *DMAS* essential for the uptake and chelation of micronutrients (Zn and Fe) in early plant growth and their redistribution to maturing seed supported by VIT activity, which is crucial for nutrient fortification within seeds. These analyses provide potential molecular targets for functional genetic research aimed at understanding micronutrient homeostasis and biofortification in wheat.

### Expression profiling of metal chelation and vacuolar sequestration pathways genes under Zn and Fe stress

The *TaNAS1*, *TaNAS6*, *TaNAS9*, *TaNAAT2*, *TaDMAS1* and *TaVIT2 genes* were selected based on their responsiveness to Zn/Fe stress, as identified through genome-wide analysis (GWA) performed in this study as well as the previously reported studies [17, 57, 58].

**Fig. 15:**
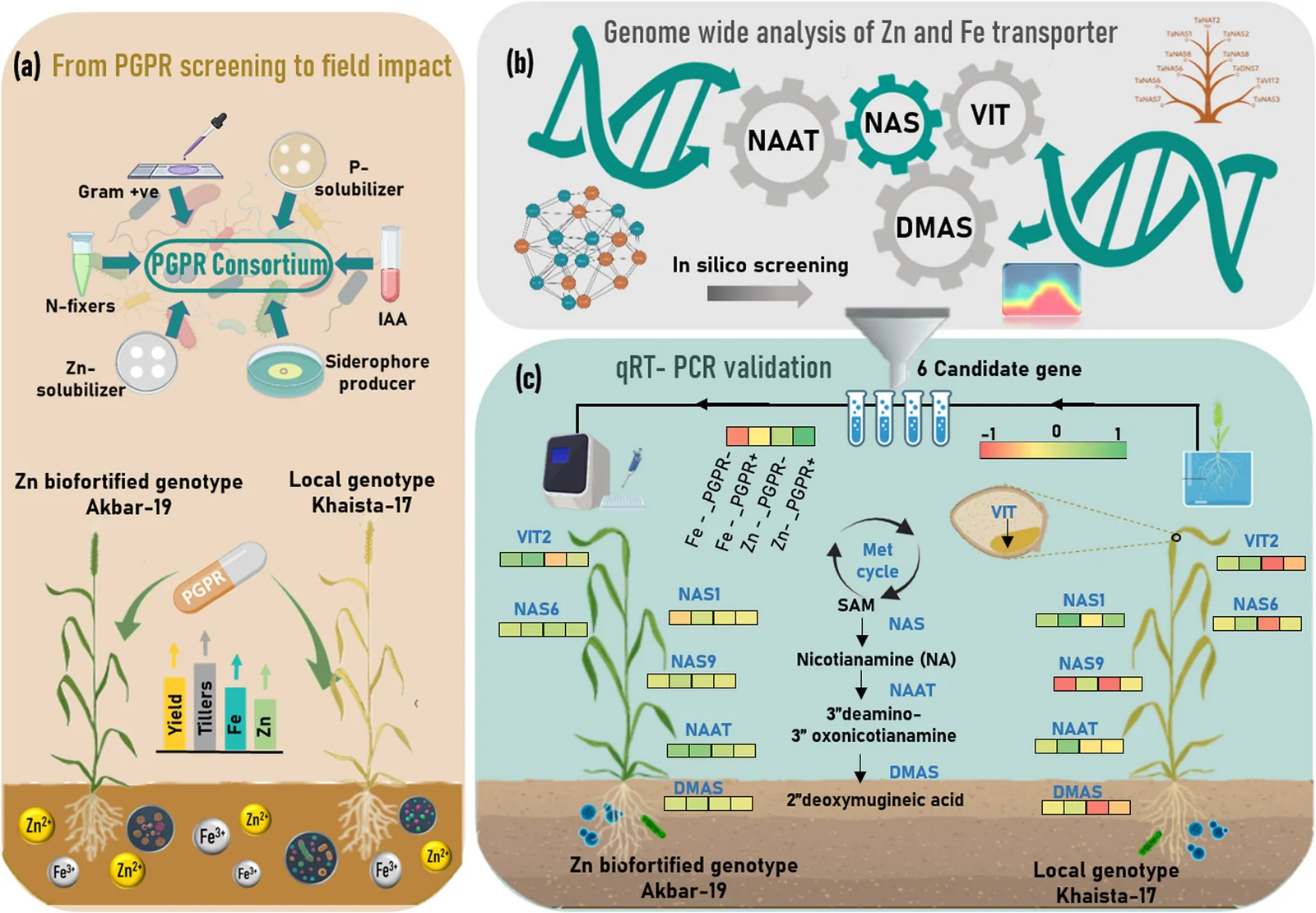
Schematic illustration of workflow. **a **From PGPR screening to field impact: PGPR characterization and its impact on agronomic traits in the studied wheat genotype (Akbar-19 and Khaista-17). **b **Genome-wide analysis of Zn and Fe transporter: In-silico analysis of NAS, NAAT, DMAS, and VIT gene families in wheat. **c **qRT-PCR validation: The expression pattern of 6 candidate genes selected from genome-wide analysis and further studied in Akbar-19 and Khaista-17. The transcript expression is depicted in a color scale. The color scale represents the log2 fold change values of differentially expressed genes. Red scale refers to negatively regulated genes, while green scale refers to positively regulated genes. Fe- (Fe stress), Zn- (Zn stress), RGPR- (PGPR uninoculated), and PGPR+ (RGPR inoculated)

The 3 TaNAS genes, TaNAS1, TaNAS6, and TaNAS9 exhibited significant upregulation in both varieties under Zn and Fe limiting conditions following inoculation with consortia Fig. 15 (c). This indicates the active participation of rhizobacteria in solubilization and nutrient chelation, thereby promoting enhanced NA activity for transport of divalent metal cations such as Fe^2+^ (forming Fe (II)-NA and Fe (III)-NA complexes) and Zn^2+^-NA in the shoot [60]. Additionally, the enhanced expression of *TaNAAT2* and *TaDMAS1* under Fe & Zn limiting conditions in the shoot of inoculated plants suggests increased production of DMA for micronutrient acquisition and translocation within wheat. This is in accordance with the previous findings [61], which reported enhanced expression of *SbNAS2* and *SbDMAS2*, resulting in increased PS release from roots in response to arbuscular mycorrhizal fungi (AMF) inoculation under Fe-deficient conditions in sorghum relative to the uninoculated treatment suggesting that NA is transcriptionally upregulated to facilitate Fe uptake via chelation and is involved in the transport of Fe within roots and shoots. The phytosiderophore (PS) production genes in wheat mainly involved in DMA production within the roots enhance the chelation of Fe and Zn under Fe and Zn-deficient conditions, as illustrated in Fig. 15 (c). Additionally, DMA and NA are fundamentally involved in both the short and long-distance transport of Fe (Fe ^2+^, Fe^3+^) and Zn^2+^as well as in the intra- and intercellular transport of these nutrients within the shoot of the wheat plant [59].

In our study, the Zn-efficient wheat variety Akbar-19 exhibited significantly enhanced expression levels of genes involved in phytosiderophore biosynthesis and metal chelation specifically NAS, NAAT, and DMAS in comparison to the Zn-inefficient cultivar under zinc-deficient conditions, suggesting an evolved molecular mechanism for Zn mobilization and uptake in Akbar-19.These findings align with previous reports by [17], where the Zn and Fe-efficient wheat cultivar Narmada 195 demonstrated enhanced expression of phytosiderophore biosynthesis genes compared to the inefficient cultivar PBW 502 under nutrient-limiting conditions. Overall, these results indicate genotype-dependent regulation of micronutrient acquisition pathways and highlight the crucial role of phytosiderophore-mediated nutrient transport in improving Zn and Fe efficiency in wheat.

An upregulation of Vacuolar-Iron transporter-like (VTL) and VIT under Fe and Zn surplus and deficient conditions has been reported validating the active participation of these gene in long distance Zn and Fe transport and its translocation from vegetative tissues to the wheat grain [46,17,62]. Our results demonstrate enhanced regulation of TaVIT2 in inoculated plants relative to the control and uninoculated plants under Zn and Fe stress conditions which suggests that the application of bacterial consortia improve uptake of Fe and Zn, thereby facilitating the transport of excess iron into the vacuole to maintain the cellular nutrient balance, which is further redistributed based on iron demand within the plant.

## Conclusions

This study systematically evaluated the synergistic effects of PGPR and micronutrient-based integrated nutrient management (INM) strategies under field conditions, focusing on their agronomic and molecular impacts across consecutive cropping seasons. The multi-season trials confirmed the reliability, reproducibility, and field-level consistency of the treatment outcomes. Two wheat genotypes Akbar-19 (a Zn-biofortified variety) and Khaista-17 (a local cultivar) were cultivated under identical nutrient regimes to assess genotype-specific responses. Both varieties exhibited a genotype-independent improvement in agronomic traits, whereas noticable differences in nutrient uptake dynamics highlighted genotype-specific sensitivities, particularly in micronutrient assimilation.

A distinctive aspect of this research is its molecular investigation of wheat transporter genes involved in zinc (Zn) and iron (Fe) chelation, transport, and homeostasis. The study examined the expression profiles of these transporters under nutrient-deficient conditions, comparing PGPR-inoculated and uninoculated treatments. Notably, PGPR inoculation significantly upregulated all targeted transporter genes, thereby facilitating improved micronutrient uptake and translocation. This led to enhanced Zn and Fe accumulation in wheat grains across both cultivars.

By integrating agronomic performance metrics with genome-wide analysis and gene expression analysis, this research bridges a critical gap in understanding the mechanistic basis of nutrient acquisition in wheat under microbial intervention. It introduces a molecular dimension to field-based agronomic studies, an approach rarely employed in conventional nutrient management research, thereby providing valuable insights into the regulation of micronutrient transporters and their role in sustainable crop biofortification.

### Research implications

1. The research highlights the possibility of using the bacterial bioformulations in combination with micronutrient fertilization at lower NPK doses as sustainable farming strategy.
2. The approach has environmental and economic advantages since it reduces the inputs of synthetic fertilizers without decreasing the yields and nutritional quality of wheat.
3. The increased grain Zn and Fe content can help eliminate Zn malnutrition in population that relies on wheat as a staple food, thereby supporting biofortification objectives at the field level.
4. The genotype-specific response demand specific strategies that maximize nutrient uptake and crop yield.

## Supplementary Information

Supplementary Material 1.

## Data Availability

The genomic and protein sequences used and/or analyzed under the current study were retrieved from Ensembl Plants and Phytozome, and the gene IDs, along with a persistent active link, are included within the additional files. The RNA-seq dataset used for gene expression analysis is available on the expVIP wheat expression platform, under the accession code DRP000768, SRP028357, ERP004505, and ERP016738.

## References

[1] Cao Y, Su X, Wang J, Shao Q, Long Z, Wu Y, Ye J, Wang M, Sun C, Wang F. Global burden of zinc deficiency among children under 5 years old from 1990 to 2020. Int J Food Sci Nutr. 2025;76(4):456–65. 10.1080/09637486.2025.2496954 40288992

[2] Nnamani JE, Agu UF, Okafor BC, Aransiola MM, Ezeamii JC, Benson OC, Appiah L, Nwokedi VU. A Review on Micronutrient Malnutrition in Pregnant Women and Children Under Five Years. J Pharma Insights Res. 2025;3(3):304–16.

[3] Elegbeleye JA, Fayemi OE, Agbemavor WS, Krishnamoorthy S, Adebowale OJ, Adeyanju AA, Mkhabela B, Bamidele OP. Beyond Calories: Addressing Micronutrient Deficiencies in the World’s Most Vulnerable Communities—A Review. Nutrients. 2025;17(24):3960. 10.3390/nu17243960 PMC12735482 41470905

[4] Islam MH, Nayan MM, Jubayer A, Amin MR. A review of the dietary diversity and micronutrient adequacy among the women of reproductive age in low-and middle‐income countries. Food Sci Nutr. 2024;12(3):1367–79. 10.1002/fsn3.3855 PMC10916566 38455218

[5] Altaf MT, Liaqat W, Jamil A, Jan MF, Baloch FS, Barutçular C, Nadeem MA, Mohamed HI. Strategies and bibliometric analysis of legumes biofortification to address malnutrition. Planta. 2024;260(4):85. 10.1007/s00425-024-04504-0 39227398

[6] Gautam A, Sharma V, Chauhan C, Thakur A, Pandey MK, Priscilla K, Dagar R, Jin X, Sukla P, Kumar A. Fortifying crops with micronutrients for sustainable global nutritional security. Agron J. 2025;117(3):e70086.

[7] Mandial K, Mahajan G. Trends in Area, Production and Productivity of Wheat—A Time-Series Analysis. Int J Environ Agric Biotechnol. 2026;035:041.

[8] Iqbal MJ, Shams N, Fatima K. Nutritional quality of wheat. In: *Wheat.* IntechOpen Rijeka, Croatia; 2022.

[9] Gupta OP, Singh A, Pandey V, Sendhil R, Khan M, Pandey A, Kumar S, Hamurcu M, Ram S, Singh G. Critical assessment of wheat biofortification for iron and zinc: a comprehensive review of conceptualization, trends, approaches, bioavailability, health impact, and policy framework. Front Nutr. 2024;10:1310020. 10.3389/fnut.2023.1310020 PMC10794668 38239835

[10] Devkota M, Sileshi GW, Senthilkumar K, Broadley MR, Mutambu D, Sila A, Devkota K, Rizal G, Kihara J. Grain zinc, iron and protein concentrations of contemporary wheat cultivars fall short of targets for human health. Nat Food 2026;7:283–95. 10.1038/s43016-026-01314-3 PMC13021525 41803449

[11] Altaf MT, Liaqat W, Bedir M, Ali A, Nadeem MA, Baloch FS. Wheat biofortification: a promising approach to improve public health. Advances in wheat breeding: towards climate resilience and nutrient security. Springer; 2024. pp. 623–51.

[12] Ali M, Ahmed I, Tariq H, Abbas S, Zia MH, Mumtaz A, Sharif M. Growth improvement of wheat (Triticum aestivum) and zinc biofortification using potent zinc-solubilizing bacteria. Front Plant Sci. 2023;14:1140454. 10.3389/fpls.2023.1140454 PMC10213544 37251763

[13] Sun Z, Yue Z, Liu H, Ma K, Li C. Microbial-assisted wheat iron biofortification using endophytic Bacillus altitudinis WR10. Front Nutr. 2021;8:704030. 10.3389/fnut.2021.704030 PMC8368724 34414208

[14] Bishnoi S, Tokas J, Rani M, Sharma P. Mechanistic insights on zinc and iron interplay driving plant growth and stress responses. Cereal Res Commun 2026;54: 713–22.

[15] Kumar N, Tiwari A, Pal N, Kumar S. Genome-wide identification, characterization and relative expression analysis of putative iron homeostasis genes: NAS, NAAT, and DMAS in hexaploid wheat and its progenitors. J Cereal Sci. 2022;105:103446.

[16] Seregin IV, Kozhevnikova AD. Nicotianamine: a key player in metal homeostasis and hyperaccumulation in plants. Int J Mol Sci. 2023;24(13):10822. 10.3390/ijms241310822 PMC10341464 37446000

[17] Gupta OP, Pandey V, Saini R, Khandale T, Singh A, Malik VK, Narwal S, Ram S, Singh GP. Comparative physiological, biochemical and transcriptomic analysis of hexaploid wheat (T. aestivum L.) roots and shoots identifies potential pathways and their molecular regulatory network during Fe and Zn starvation. Genomics. 2021;113(5):3357–72. 10.1016/j.ygeno.2021.07.029 34339815

[18] Wollum A. Cultural methods for soil microorganisms. Methods soil analysis: part 2 Chem Microbiol Prop. 1982;9:781–802.

[19] Gontia-Mishra I, Sapre S, Tiwari S. Zinc solubilizing bacteria from the rhizosphere of rice as prospective modulator of zinc biofortification in rice. Rhizosphere. 2017;3:185–90.

[20] Paul D, Sinha SN. Isolation and characterization of phosphate solubilizing bacterium Pseudomonas aeruginosa KUPSB12 with antibacterial potential from river Ganga, India. Annals Agrarian Sci. 2017;15(1):130–6.

[21] Hardy RW, Holsten RD, Jackson E, Burns R. The acetylene-ethylene assay for N2 fixation: laboratory and field evaluation. Plant Physiol. 1968;43(8):1185–207. 10.1104/pp.43.8.1185 PMC1086994 16656902

[22] Schwyn B, Neilands J. Universal chemical assay for the detection and determination of siderophores. Anal Biochem. 1987;160(1):47–56. 10.1016/0003-2697(87)90612-9 2952030

[23] Gordon SA, Weber RP. Colorimetric estimation of indoleacetic acid. Plant Physiol. 1951;26(1):192. 10.1104/pp.26.1.192 PMC437633 16654351

[24] Irabor A, Mmbaga M. Evaluation of Selected Bacterial Endophytes for Biocontrol Potential against Phytophthora Blight of Bell Pepper (Capsicum annuum L.). J Plant Pathol Microbiol. 2017;8:424.

[25] Singh D, Geat N, Rajawat MVS, Mahajan MM, Prasanna R, Singh S, Kaushik R, Singh RN, Kumar K, Saxena AK. Deciphering the mechanisms of endophyte-mediated biofortification of Fe and Zn in wheat. J Plant Growth Regul. 2018;37(1):174–82.

[26] Zulfiqar U, Hussain S, Maqsood M, Ishfaq M, Ali N. Zinc nutrition to enhance rice productivity, zinc use efficiency, and grain biofortification under different production systems. Crop Sci. 2021;61(1):739–49.

[27] Hu S, Du B, Mu G, Jiang Z, Li H, Song Y, Zhang B, Xia J, Rouached H, Zheng L. The transcription factor OsbZIP48 governs rice responses to zinc deficiency. Plant Cell Environ. 2024;47(5):1526–42. 10.1111/pce.14825 38251320

[29] Gupta OP, Singh A, Pandey V, Sendhil R, Khan MK, Pandey A, Kumar S, Hamurcu M, Ram S, Singh G. Critical assessment of wheat biofortification for iron and zinc: a comprehensive review of conceptualization, trends, approaches, bioavailability, health impact, and policy framework. Front Nutr. 2024;10:1310020. 10.3389/fnut.2023.1310020 PMC10794668 38239835

[30] Khatri A, Bhateria R. Efficacy of nanofertilizers over chemical fertilizers in boosting agronomic production. Nat Environ Pollution Technol. 2023;22(2):767–76.

[31] Hasan A, Tabassum B, Hashim M, Khan N. Role of plant growth promoting rhizobacteria (PGPR) as a plant growth enhancer for sustainable agriculture: A review. Bacteria. 2024;3(2):59–75.

[32] Ahmad M, Hussain A, Dar A, Luqman M, Ditta A, Iqbal Z, Ahmad HT, Nazli F, Soufan W, Almutairi K. Combating iron and zinc malnutrition through mineral biofortification in maize through plant growth promoting Bacillus and Paenibacillus species. Front Plant Sci. 2023;13:1094551. 10.3389/fpls.2022.1094551 PMC9929565 36816488

[33] Jalal A, Oliveira CES, Bastos AC, Fernandes GC, De Lima BH, Furlani Junior E, De Carvalho PHG, Galindo FS, Gato IMB. Teixeira Filho MCM: Nanozinc and plant growth-promoting bacteria improve biochemical and metabolic attributes of maize in tropical Cerrado. Front Plant Sci. 2023;13:1046642. 10.3389/fpls.2022.1046642 PMC9878843 36714773

[34] Khalili A, Qayyum A, Khan SU, Ullah I, Khalofah A. Role of temporal Zn fertilization along with Zn solubilizing bacteria in enhancing zinc content, uptake, and zinc use efficiency in wheat genotypes and its implications for agronomic biofortification. Agronomy. 2023;13(11):2677.

[35] Ali M, Sharif M, Ahmad W, Ahmed I. Assessing potent zinc solubilizing bacteria to augment wheat yield and zinc biofortification. Gesunde Pflanzen. 2023;75(4):1061–73.

[36] Yadav RC, Sharma SK, Varma A, Singh UB, Kumar A, Bhupenchandra I, Rai JP, Sharma PK, Singh HV. Zinc-solubilizing Bacillus spp. in conjunction with chemical fertilizers enhance growth, yield, nutrient content, and zinc biofortification in wheat crop. Front Microbiol. 2023;14:1210938. 10.3389/fmicb.2023.1210938 PMC10352851 37469421

[37] Galindo FS, Rodrigues WL, Fernandes GC, Boleta EHM, Jalal A, Rosa PAL, Buzetti S, Lavres J, Teixeira Filho MCM. Enhancing agronomic efficiency and maize grain yield with Azospirillum brasilense inoculation under Brazilian savannah conditions. Eur J Agron. 2022;134:126471.

[38] Jalal A, Oliveira CES, Fernandes GC, da Silva EC, da Costa KN, de Souza JS, Leite GS, Biagini ALC, Galindo FS, Teixeira Filho MCM. Integrated use of plant growth-promoting bacteria and nano-zinc foliar spray is a sustainable approach for wheat biofortification, yield, and zinc use efficiency. Front Plant Sci. 2023;14:1146808. 10.3389/fpls.2023.1146808 PMC10200892 37223804

[39] Ahmad J, Tanveer SK, Nadeem M, Farooq U, Hussain F. Success Story: Akbar-19 High Zinc and High Yielding Wheat Variety of Pakistan. In: Tanveer SK, Rashid MH, Razzaque MA, editors. Dev Dissemination Biofortified varieties different crops SAARC Memb States: SAARC Agric Centre; 2024. p.78- 89.

[40] Ullah I, Haneef Raza SA, Fahad S, Ahmad A, Mukhtiar S, Samad N, Babar M. 11. Stability and yield performance of different wheat genotypes across multi-locations of Khyber Pakhtunkhwa. Pure Appl Biology (PAB). 2022;11(3):793–805.

[41] Kukreti B, Singh AV. Unlocking the biofortification potential of Serratia marcescens for enhanced zinc and iron content in wheat grains. Pantnagar J Res. 2024;22:1.

[42] Khan ST, Malik A, Alwarthan A, Shaik MR. The enormity of the zinc deficiency problem and available solutions; an overview. Arab J Chem. 2022;15(3):103668.

[43] Shakeel M, Hafeez FY, Malik IR, Rauf A, Jan F, Khan I, Ijaz I, Elsadek MF, Ali MA, Rashid K. Zinc solubilizing bacteria synergize the effect of zinc sulfate on growth, yield and grain zinc content of rice (Oryza sativa). Cereal Res Commun. 2024;52(3):961–71.

[44] Lilay GH, Thiébaut N, du Mee D, Assunção AG, Schjoerring JK, Husted S, Persson DP. Linking the key physiological functions of essential micronutrients to their deficiency symptoms in plants. New Phytol. 2024;242(3):881–902. 10.1111/nph.19645 38433319

[45] Roy C, Kumar S, Ranjan RD, Kumhar SR, Govindan V. Genomic approaches for improving grain zinc and iron content in wheat. Front Genet. 2022;13:1045955. 10.3389/fgene.2022.1045955 PMC9683485 36437911

[46] Sharma S, Kaur G, Kumar A, Meena V, Ram H, Kaur J, Pandey AK. Gene expression pattern of vacuolar-iron transporter-like (VTL) genes in hexaploid wheat during metal stress. Plants. 2020;9(2):229. 10.3390/plants9020229 PMC7076494 32053953

[47] Gautam T, Jan I, Batra R, Singh K, Pandey R, Sharma PK, Balyan HS, Gupta PK. Further studies on nicotianamine aminotransferase (NAAT) genes involved in biofortification in bread wheat (Triticum aestivum L). Plant Gene. 2023;33:100389.

[48] Liu G, Sun Y, He P, Wang Y, Zhang Y, Li Q, Liu F, Zhang S, Zhang Y, Liu C. Identification of the Nicotianamine synthase (NAS) gene family in wheat (Triticum aestivum L.) and the role of its member TaNAS4-A in Zn and Fe transport. Funct Integr Genom. 2026;26(1):11. 10.1007/s10142-025-01798-5 41432786

[49] Bonneau J, Baumann U, Beasley J, Li Y, Johnson AA. Identification and molecular characterization of the nicotianamine synthase gene family in bread wheat. Plant Biotechnol J. 2016;14(12):2228–39. 10.1111/pbi.12577 PMC5103229 27155533

[50] Sharma S, Kaur G, Kumar A, Meena V, Kaur J, Pandey AK. Overlapping transcriptional expression response of wheat zinc-induced facilitator-like transporters emphasize important role during Fe and Zn stress. BMC Mol Biol. 2019;20(1):22. 10.1186/s12867-019-0139-6 PMC6757437 31547799

[51] De Abreu KE. CRISPR-Cas9 gene editing provides evidence for a role of F-group bZIP transcription factors in Zn homeostasis in barley. University of Southampton; 2025.

[52] Zhang X, Xiao K, Li S, Li J, Huang J, Chen R, Pang S, Zhou X. Genome-wide analysis of the NAAT, DMAS, TOM, and ENA gene families in maize suggests their roles in mediating iron homeostasis. BMC Plant Biol. 2022;22(1):37. 10.1186/s12870-021-03422-7 PMC8762928 35039017

[53] Connorton JM, Jones ER, Rodríguez-Ramiro I, Fairweather-Tait S, Uauy C, Balk J. Wheat vacuolar iron transporter TaVIT2 transports Fe and Mn and is effective for biofortification. Plant Physiol. 2017;174(4):2434–44. 10.1104/pp.17.00672 PMC5543970 28684433

[54] Zhang Y, Guan J, Chai T, Qiao K. A biofortification tool enhances iron and manganese tolerance and accumulation in plants. J Sci Food Agric. 2025;105(14):8296–306. 10.1002/jsfa.70076 40686439

[55] Agrwal K, Veena P, Singhal N, Pandey AK. Vacuolar iron transporter-like protein VTL5 enhances iron and zinc accumulation in wheat grain endosperm. Plant Biotechnol J. 2025;23(10):4503. 10.1111/pbi.70227 PMC12483938 41028990

[56] Nasim A, Hao J, Tawab F, Jin C, Zhu J, Luo S, Nie X. Micronutrient biofortification in wheat: QTLs, candidate genes and molecular mechanism. Int J Mol Sci. 2025;26(5):2178. 10.3390/ijms26052178 PMC11900071 40076800

[57] Beasley JT, Bonneau JP, Johnson AA. Characterisation of the nicotianamine aminotransferase and deoxymugineic acid synthase genes essential to Strategy II iron uptake in bread wheat (Triticum aestivum L). PLoS ONE. 2017;12(5):e0177061. 10.1371/journal.pone.0177061 PMC5419654 28475636

[58] Bonneau J, Baumann U, Beasley J, Li Y, Johnson AAT. Identification and molecular characterization of the nicotianamine synthase gene family in bread wheat. Plant Biotechnol J. 2016;14(12):2228–39. 10.1111/pbi.12577 PMC5103229 27155533

[59] Ning X, Lin M, Huang G, Mao J, Gao Z, Wang X. Research progress on iron absorption, transport, and molecular regulation strategy in plants. Front Plant Sci. 2023;14:1190768. 10.3389/fpls.2023.1190768 PMC10351017 37465388

[60] Dhankhar R, Gupta S, Gulati P. Insights on plant–microbe interactions in soil in relation to iron dynamics. Vegetos. 2023;36(3):750–67.

[61] Prity SA, Sajib SA, Das U, Rahman MM, Haider SA, Kabir AH. Arbuscular mycorrhizal fungi mitigate Fe deficiency symptoms in sorghum through phytosiderophore-mediated Fe mobilization and restoration of redox status. Protoplasma. 2020;257(5):1373–85. 10.1007/s00709-020-01517-w 32535729

[62] Kenzhebayeva S, Atabayeva S, Sarsu F, Abekova A, Shoinbekova S, Omirbekova N, Doktyrbay G, Beisenova A, Shavrukov Y. Organ-specific expression of genes involved in iron homeostasis in wheat mutant lines with increased grain iron and zinc content. PeerJ. 2022;10:e13515. 10.7717/peerj.13515 PMC9190668 35707120

[63] Ain Qu, Hussain HA, Zhang Q, Maqbool F, Ahmad M, Mateen A, Zheng L,Imran A. Coordinated influence of Funneliformis mosseae and different plantgrowth-promoting bacteria on growth, root functional traits, and nutrientacquisition by maize. Mycorrhiza. 2024;34(5):477–88. 10.1007/s00572-024-01165-5 39115556

[64] Mirza MS, Mehnaz S, Normand P, Prigent-Combaret C, Moënne-LoccozY, Bally R, Malik KA. Molecular characterization and PCR detection of anitrogen-fixing Pseudomonas strain promoting rice growth. Biol Fertil Soils.2006;43(2):163–70.

[65] Zaheer A, Mirza BS, Mclean JE, Yasmin S, Shah TM, Malik KA, Mirza MS.Association of plant growth-promoting Serratia spp. with the root nodules ofchickpea. Res Microbiol. 2016;167(6):510–20. 10.1016/j.resmic.2016.04.001 27117242

